# Natural Microbial Communities Can Be Manipulated by Artificially Constructed Biofilms

**DOI:** 10.1002/advs.201901408

**Published:** 2019-09-19

**Authors:** Tomaž Rijavec, Jan Zrimec, Rob van Spanning, Aleš Lapanje

**Affiliations:** ^1^ Department of Environmental Sciences Jožef Stefan Institute Jamova cesta 39 1000 Ljubljana Slovenia; ^2^ Institute of Metagenomics and Microbial Technologies Clevelandska ulica 19 1000 Ljubljana Slovenia; ^3^ Systems and Synthetic Biology Chalmers University of Technology Kemivägen 10 412 96 Göteborg Sweden; ^4^ Systems Bioinformatics Faculty of Science Vrije Universiteit Amsterdam De Boelelaan 1105 1081 HV Amsterdam The Netherlands

**Keywords:** 16S rRNA, bacteria, layer‐by‐layer (LBL), metagenomic, nanolayers, polyelectrolytes

## Abstract

Biofouling proceeds in successive steps where the primary colonizers affect the phylogenetic and functional structure of a future microbial consortium. Using microbiologically influenced corrosion (MIC) as a study case, a novel approach for material surface protection is described, which does not prevent biofouling, but rather shapes the process of natural biofilm development to exclude MIC‐related microorganisms. This approach interferes with the early steps of natural biofilm formation affecting how the community is finally developed. It is based on a multilayer artificial biofilm, composed of electrostatically modified bacterial cells, producing antimicrobial compounds, extracellular antimicrobial polyelectrolyte matrix, and a water‐proof rubber elastomer barrier. The artificial biofilm is constructed layer‐by‐layer (LBL) by manipulating the electrostatic interactions between microbial cells and material surfaces. Field testing on standard steel coupons exposed in the sea for more than 30 days followed by laboratory analyses using molecular‐biology tools demonstrate that the preapplied artificial biofilm affects the phylogenetic structure of the developing natural biofilm, reducing phylogenetic diversity and excluding MIC‐related bacteria. This sustainable solution for material protection showcases the usefulness of artificially guiding microbial evolutionary processes via the electrostatic modification and controlled delivery of bacterial cells and extracellular matrix to the exposed material surfaces.

## Graphical abstract

**The artificial biofilm** is a multilayered structure composed of the selected bacterial cells, extracellular polyelectrolyte matrix, and rubber elastomer. It is constructed on the surface of materials layer‐by‐layer conditioning it physically, chemically, and biologically. Its application manipulates the processes of biofouling and the development of natural microbial communities by excluding corrosion causing microorganisms.

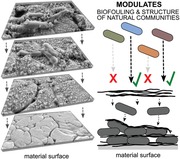

## 1 Introduction

Corrosion of carbon steel in seawater proceeds in two‐stages. The first, aerobic stage depends on oxygen availability, is abiotic, and is characterized by the fast corrosion rate. The second, anaerobic stage has a slower corrosion rate and is mainly microbiologically influenced (reviewed in^1^). In this stage the corrosion‐related microorganism oxidize Fe^2+^ to Fe^3+^ directly or by producing H_2_S, O_2_, chelators and organic acids (reviewed in^2^, ^3^). The microbiologically‐influenced corrosion (MIC) is difficult to combat due to the complex environmental conditions that chemically modify the properties of the engineered surfaces and the MIC‐related bacterial groups, which are phylogenetically and physiologically extremely diverse and are thus quick to adapt to different antimicrobial, antiattachment, and microbe‐repelling MIC preventing strategies.^4^

The currently available corrosion‐prevention solutions primarily target the abiotic aerobic stage by establishing a water barrier offering chemical inhibition and/or galvanic protection. The solutions include organic, metallic and nonmetallic inorganic coatings,^5^ of which, the former also incorporate biocidal or antifouling biomimetic additives.^6^, ^7^ Regardless of the formulation, all available solutions eventually fail, since: i) barrier coatings lose their structural integrity on the long run, ii) biocide additives are not universal inhibitors of microorganisms and iii) biocide action is time limited and toxic to the environment (reviewed in^8^).

Even though anticorrosion solutions were developed to kill or mitigate microbial attachment, it is well known that biofouling, a prerequisite for the initiation of MIC, cannot be stopped or avoided. However, some biofilm forming bacteria can inhibit corrosion in vitro (reviewed in^9^ by: i) consuming oxygen,^10^ ii) producing an extracellular polymer matrix,^11^ iii) producing antimicrobials against corrosion‐causing microbes, such as gramicidin S,^12^ or iv) biocompetitive exclusion.^13^ We therefore propose that novel sustainable solutions for material protection must not focus merely on the prevention of biofouling, but rather on shaping the natural process of biofilm development to exclude MIC‐related microorganisms. This concept is new to the field of corrosion protection, but the idea to modulate the structure of microbiomes has been discussed widely for probiotic bacteria in relation to the human gut,^14^, ^15^ skin wounds,^16^ the oral cavity,^17^ and agricultural systems.^18^ In the environment the biofouling process consist of individual colonization steps, which are influenced by the previously established surface microbiota.^19^ Since the initial physicochemical conditions and primary microbial colonizers play a major part in determining the composition of a future microbial community (reviewed in^20^, ^21^, ^22^), the natural development of corrosion‐causing biofilms can be manipulated by priming the surface of metals with the extracellular matrix and bacteria of our choice.

Since one cannot simply force the bacterial cells to attach and because random deposits on the metal surface are not useful, construction of structured artificial biofilms offers an efficient alternative. The artificial biofilm is constructed on the metal surface in multiple layers and sets the initial physicochemical and biological conditions by integrating a conditioning film^23^ and an extracellular matrix made of polyelectrolytes (PEs) with bacterial cells and a water‐barrier coating^24^ made of a rubber elastomer. The layer‐by‐layer (LBL) approach, which was originally implemented in colloid physics, allows the deposition of each layer of the biofilm using PEs and electrostatic interactions between the layers. (reviewed in^25^, ^26^). PEs with opposite charges are applied to surfaces of materials and cells making the two stick to each other. When multiple layers of PEs are deposited over the surface of cells, the cells become entrapped within a nanometer thick PE‐based LBL capsule.^25^ The charged PEs of the capsule can be used to deposit the cells to a material surface via electrostatic interactions,^27^, ^28^ which can help the cells spread over the surface after breaking free. Alternatively, the capsule can be used for slow release of antimicrobial compounds^29^, ^30^ after the cell had died. Studies on LBL modifications of cell surfaces have most commonly implemented artificial PEs, such as poly(styrenesulfonate) (PSS) or poly(acrylic acid) (PAA),^26^, ^31^, ^32^ or natural PEs, such as chitosan,^33^ which themselves show antimicrobial properties. The LBL coated surfaces can easily be combined with rubber elastomers, following the same principles of electrostatic interactions between the deposited layers. Rubber elastomers add a water barrier property to the final solution, are simple to apply by spray deposition and drying, and do not need special physical or chemical curing (e.g., Plastidip rubber elastomer^34^, ^35^). To date, they have not been implemented for solutions, such as the one described in this study.

With the goal to shape the development of natural biofilms that form on the surface of steel during exposure in seawater, we constructed a protective multilayer artificial biofilm, incorporating three main components: i) the extracellular anticorrosive and antibacterial PE matrix composed of artificial and natural PEs, ii) the bacterial cells producing antibacterial compounds, and iii) the water‐barrier consisting of rubber elastomers. The artificial biofilm was structured such that the gramicidin S producing bacterial cells of *Brevibacillus brevis*, encapsulated with artificial PEs, were enclosed within the rubber elastomer water‐resistant layer and were thus not directly in contact with the environment, while the natural PE matrix and the antimicrobial‐producing cells of *Bacillus pumilus*, surface‐modified with natural PEs, were deposited on the surface of the rubber elastomer layer, to be in direct contact with the environment. We then performed a field test in natural seawater and examined by 16S rRNA metagenetic analysis how the composition of the natural bacterial community was modified when the artificial biofilm was applied to the steel surface. An expected shift in the phylogenetic composition of the community would indicate that the bacterial groups related to MIC were excluded during the biofouling process.

## 2 Results

### 2.1 Electrostatic Modification of Cells and Metal Surfaces

**Figure 1:**
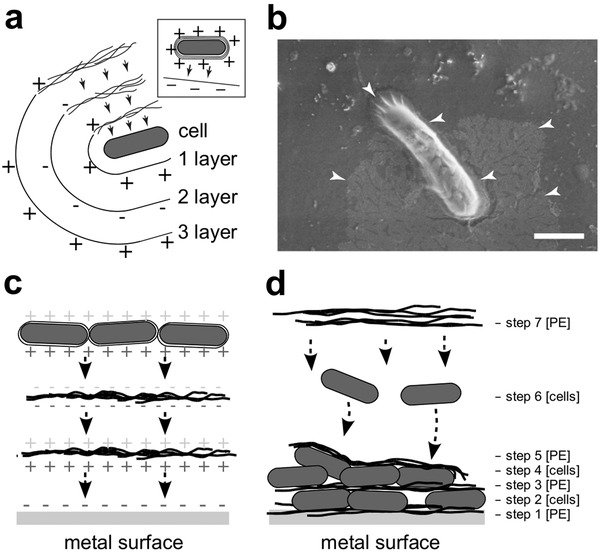
The electrostatic modification of surfaces using charged polyelectrolytes (PEs). The layer‐by‐layer (LBL) approach is used to modify the surfaces of cells and materials electrostatically. Oppositely charged polyelectrolytes are deposited over each other in consecutive layers. The final layer determines the charge. This helps in depositing the cells to oppositely charged surfaces to construct an artificial biofilm structure. The cells are represented as round gray oval shapes and the polyelectrolytes by black freehand lines. (+/−) designates the charges of PEs or the modified surfaces of cells or metal. a) The LBL electrostatic modification of cells allows the construction of a PE capsule around the cell. b) The positively charged capsule forces the cell to stick to the negatively charged abiotic surface. White arrowheads denote PE, which is seen as a veil‐like structure attaching the cell to the surface of the material. c) The LBL electrostatic modification of a metal surface uses the same principle layer after layer. d) A multilayer structure can be constructed on the metal surface using charged PEs and surface‐modified cells.

Using charged PEs, we electrostatically modified the surfaces of bacterial cells (**Figure** **1**): i) to construct a PE capsule around the cell, controlling the diffusion of bacterial secondary metabolites and ii) to successfully deposit bacterial cells onto the metal surface by increasing their attraction to the oppositely charged surface. The initial ζ‐potential measurement of the unmodified cells of the stationary cell culture (OD_600_ > 1.3) indicated that the surfaces of native cells were negative, but not equal between the two selected strains DSM30 and DEV1 (**Tables** **1** and **2**). A single washing step with 0.9% NaCl was sufficient to equalize the ζ‐potentials of both strains to approximately −34 mV. Successful electrostatic deposition of the first, positively charged PE, shifted the ζ‐potential values to positive and vice versa, when negatively charged PE was deposited on top of the first layer (Tables 1 and 2). The deposition of the third and final, positive PE layer, again switched the ζ‐potential to positive (+20.8 ± 0.9 mV; Table 2).

**Table 1:** The ζ‐potential of cells of *B. brevis* strain DSM 30. The ζ‐potential of cells changes according to the charge of the polyelectrolyte that is deposited as the last layer. The ζ‐potential is used as indicator of surface charge

	Unmodified cells (fresh culture)	Unmodified cells (1× wash)^a)^	Unmodified cells (3× wash)^a)^	LBL modified cells (1 layer)^b)^	LBL modified cells (2 layers)^c)^, ^d)^	LBL modified cells (3 layers)^e)^	LBL modified cells (4 layers)^f)^
ζ‐potential	−17.3 ± 0.5	−33.8 ± 0.8	−33.1 ± 0.9	+17.99 ± 0.70	−35.58 ± 1.38	+19.33 ± 1.39	−40.48 ± 1.82

^a)^ Washing is carried out by resuspending the cells in an equal volume of sterile 0.9% NaCl

^b)^ Cell surface is covered by a single layer of the positively charged polyethylenimine (PEI[+])

^c)^ Cells are covered by two layers of polyelectrolytes, first PEI[+], followed by the negatively charged PSS[−] as the top layer

^d)^ A similar shift to a negative value, was observed when PAA[−] was used for deposition of the second layer instead of PSS. ζ‐potential was determined to be −50.31 ± 8.15. PAA was used in the final solution due to its higher succeptibility to biological degradation^36^

^e)^ Cells are covered by three layers of polyelectrolytes, PEI[+], PSS[−], and once again PEI[+] as the top layer

^f)^ Cells are covered by four layers of polyelectrolytes, PEI[+], PSS[−], PEI[+], and once again PSS[−] as the top layer.

**Table 2:** The ζ‐potential of cells of the environmental strain DEV1. The ζ‐potential of cells changes according to the charge of the polyelectrolyte deposited to their surface last. The ζ‐potential is used as indicator of surface charge

	Unmodified cells (fresh culture)	Unmodified cells (1× wash)^a)^	Unmodified cells (3× wash)^a)^	Modified cells (3‐layer capsule)^b)^
ζ‐potential	−36.9 ± 1.0	−33.0 ± 1.4	−34.3 ± 0.5	+20.8 ± 0.9

^a)^ Washing is carried out by resuspending the cells in an equal volume of sterile 0.9% NaCl

^b)^ LBL nanocapsule consisting of 3 consecutive layers of charged polyelectrolytes (chitosan[+]–lignosulphonate[−]– chitosan[+]).

The surface ζ‐potential of raw unmodified steel was measured as slightly negative (−0.6 ± 0.3 mV; **Table** **3**). The negative potential increased by the application of the Plasti Dip adhesion primer used as the first modification layer (−57.0 ± 0.6 mV). The following depositions of, first, alumina and second, lignosulphonate shifted the ζ‐potential to positive and back to negative, respectively (Table 3). Further deposition of each charged layer resulted in either positive or negative surface ζ‐potential (data not shown) and was finally measured as −6.3 ± 0.5 mV after the deposition of a negative layer of lignosulfonate as the tenth layer (Table 3).

**Table 3:** The ζ‐potential of the modified metal surface. The ζ‐potential of the metal surface changes according to the charge of the polyelectrolyte deposited last. The ζ‐potential is used as indicator of surface charge

	Raw steel surface (Q‐Pannel, R46)	Adhesion primer (Plasti Dip)	1 layer LBL modification^a)^	3 layer LBL modification^b)^	10 layer LBL modification^c)^
Surface ζ‐potential	−0.6 ± 0.3	−57.0 ± 0.6	+35.7 ± 0.9	+30.0 ± 1.0	−6.3 ± 0.5

^a)^ Single layer modification of the surface by the positively charged alumina nanowires

^b)^ 3‐layer polyelectrolyte coating prepared on the metal surface in consecutive layers (alumina[+]–lignosulfonate[−]–alumina[+]). The positively charged alumina is used as top layer

^c)^ 10‐layer polymer structure prepared on the metal surface with alternating layers of alumina[+] and lignosulphonate[−]. Negatively charged lignosulphonate is used as top layer.

### 2.2 Deposition of Modified Cells to Test Surfaces

To examine the attachment of the electrostatically modified cells (Figure 1) to oppositely charged test surfaces of glass and metal we modified the surface of cells of strain *B. pumilus* DSM 30 by PEI (+18.0 ± 0.7 mV) and characterized the ζ‐potential of the surface of glass slides (−67.7 ± 1.6 mV) and the R‐46 steel coupons covered by the Plasti Dip rubber elastomer (−61.8 ± 1.7 mV). Using phase‐contrast microscopy and fluorescence microscopy to visualize the surface of glass (Figure S1a,c, Supporting Information) and steel (Figure S1b,d, Supporting Information), respectively, we confirmed that the cells deposited onto the surfaces were not washed off by rinsing the surface with water and that the surface area covered by the cells reflected the concentration of cells in the initial suspension (Figure S1, Supporting Information). Depositing PE‐encapsulated positively charged single cells to a clean surface of the negatively charged rubber elastomer, covering the surface of steel, demonstrated how the PE assured the attachment of cells to the oppositely charged surface. The PE formed a veil‐like structure enclosing the cell, as well as covering the surface of the material around the cell (Figure 1b).

### 2.3 Deposition of Individual Layers of the Artificial Biofilm

**Figure 2:**
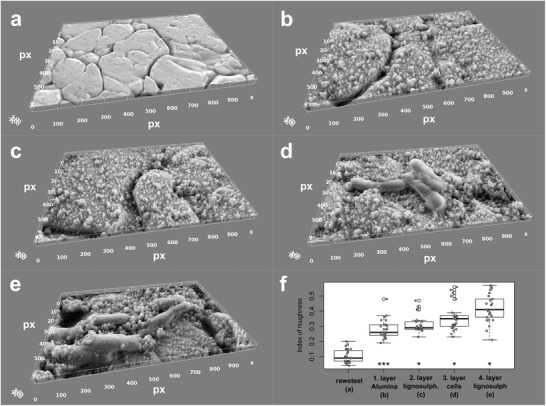
Visualization of the deposition of the first 4 layers of the artificial biofilm on stainless steel. Each deposited layer is visualized by SEM and its surface roughness is calculated by image analysis. a) Unmodified raw surface of stainless steel, b) the positively charged alumina nanowires deposited as the first layer, c) the negatively charged lignosulphonate deposited as the second layer, d) the positively charged, LBL‐modified cells deposited as the third layer, and e) the negatively charged lignosulfonate deposited over the cells as the fourth layer. f) The calculated roughness of the surface is presented as the coefficient of variation of gray values on random 25 subsamples of the SEM image (see the Experimental Section). Statistical comparison of datasets was performed using Mann–Whitney test; *, *p* < 0.05; **, *p* < 0.01; ***, *p* < 0.001. The dataset of each layer is compared to the dataset of the preceding layer.

**Figure 3:**
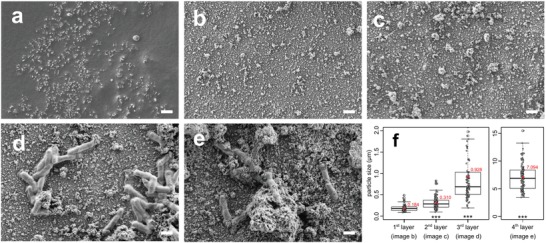
Visualization of the deposition of the first four layers of artificial biofilm on the surface of the rubber elastomer coating. Each deposited layer is visualized by SEM. a) The unmodified surface of the rubber elastomer coating covering the R‐46 steel. b) The positively charged alumina nanowires are deposited over the negatively charged surface of the rubber elastomer. c) The negatively charged layer of lignosulphonate is deposited over the positively charged layer of alumina. d) The positively charged LBL‐encapsulated cells are deposited over the first two layers. e) The fourth layer of the negatively charged lignosulphonate is deposited over the other three layers. f) The size of the particles on the surface increases as each consecutive layer is deposited over the surface. Scale (white line): 1 µm. Statistical comparison of datasets was performed using Mann–Whitney test; ***, *p* < 0.001. The dataset of each layer is compared to the dataset of the preceding layer.

Using scanning electron microscopy (SEM), we monitored the deposition of each of the first four layers, consisting of either a PE or LBL‐modified bacterial cells, onto the surface of i) stainless steel (Q‐panel SS‐36; Q‐Lab, Germany) (**Figure** **2**) and ii) standard steel (Q‐panel R‐46) precoated by Plastidip primer and rubber elastomer (**Figure** **3**). Among the deposited layers, which all increased the roughness of the surface (Figure 2f), the first layer (alumina nanowires), was the most prominent in doing so (*p* < 0.001; Mann–Whitney test). Each layer increased the size and changed the shape of the deposited particles. The first two, abiotic layers, the positively charged alumina nanowires and the negatively charged lignosulphonate, were observed as an evenly distributed granulated deposit (Figures 2b,c, and 3b,c) with average size bellow 0.5 µm (Figure 3f). Alternatively, the encapsulated bacterial cells were observed as large rod shaped particles with the average length 1.92 ± 0.43 µm (*n* = 10) and width 0.54 ± 0.07 µm (*n* = 10), deposited separately or in clumps (Figures 1b, 2d, and 3d), making the average size of all the deposited particles around 1 µm in diameter (Figure 3f). The following layer of the positively charged lignosulphonate deposited on top of the bacterial cells (Figures 2e and 3e) further increased the granulation of the surface, making the average size of the granules well above 1 µm in diameter (Figure 3f).

### 2.4 The Artificial Biofilm and the Surfaces Used as Controls

To carry out the field test in seawater, we successfully constructed a multilayered artificial biofilm on the surface of the R‐46 steel coupons, incorporating i) the water‐resistant rubber elastomer, ii) natural and artificial PEs forming the extracellular matrix, and iii) two types of LBL encapsulated bacterial cells producing antimicrobial compounds. In the artificial biofilm structure, the Plasti Dip rubber elastomer divided the bottom and upper sections, each composed of its characteristic PEs and bacterial strains (Table S1, Supporting Information). The bottom section incorporated the positively charged alumina nanowires, the negatively charged lignosulfonate and the PEI/PAA encapsulated cells of strain DSM30 and was constructed on the surface of the preapplied Plasti Dip primer coating. The upper section incorporated the positively charged chitosan, the negatively charged lignosulfonate and the chitosan/lignosulfonate encapsulated cells of strain DEV1 and was constructed on top of the Plasti Dip rubber elastomer dividing layer. In the field experiment, raw unmodified R‐46 steel coupons (control surface I) and coupons coated only with the Plasti Dip rubber elastomer (control surface II) were used as controls (see the Experimental Section for details).

### 2.5 The Artificial Biofilm Changes the Temporal Development of Natural Communities

**Figure 4:**
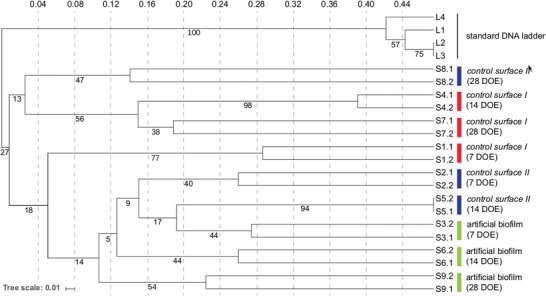
DGGE analysis of the temporal development of bacterial communities on the modified surfaces exposed to seawater in Piran, Slovenia. Raw untreated R‐46 steel (control surface I), steel coated with the rubber elastomer (control surface II), and steel coated with the artificial biofilm solution (artificial biofilm) were used for comparison. DGGE profiles of the communities were examined on both sides of the test coupons (S*x*.1, S*x*.2) at 7, 14, and 28 days of exposure (DOE). Tree parameters used are unweighted pair group method with arithmetic mean and Jaccard's coefficient of similarity. Tree scale represents distance. Numbers on tree represent bootstrap values calculated for 1000 repetitions.

In the field experiments in seawater, the temporal development of natural bacterial communities on the exposed surfaces were examined by the accumulated microbial biomass on the surface (Table S2, Supporting Information) and by examining the 16S‐rRNA‐based phylogenetic profiles using denaturing gradient gel electrophoresis (DGGE) (**Figure** **4**). During the full 42 days of exposure (DOE) in the seawater of the Gulf of Napoly, Italy, the control unmodified raw surface of steel (control surface I) accumulated the highest microbial biomass (100 times higher content of total DNA) compared to the other two samples, while showing the lowest accumulation in the early period up to 28 DOE in the seawater in Piran, Slovenia. Steel protected by the rubber elastomer (control surface II) and the artificial biofilm solution showed comparable levels throughout the exposition period at both test sites, with the lowest levels at 42 DOE. As observed in the first field experiment the phylogenetic patterns of the developing communities in the time period 0 to 28 DOE, reflected the type of the exposed surface (Figure 4), where the raw metal surface (control surface I) was clearly distinct from both of the two treatments (control surface II and artificial biofilm) (*p* < 0.01; Unifrac test), while the distinction between the two treatments was not as obvious (*p* = 0.56; Unifrac test). The communities of all three sample types were the most similar among each other at the early 7 DOE with their similarity decreasing temporally. The raw steel (control surface I), at 14 and 28 DOE, and the rubber elastomer (control surface II), at 28 DOE, thus formed a separate cluster, while the artificial biofilm solution, at 28 DOE, still resembled its earlier temporal stages (7 and 14 DOE), meaning that the community, influenced by the artificial biofilm solution, stayed the most uniform throughout the test period. A comparison of communities on both sides of the test coupon were confirmed to be uniform in all samples analyzed (*p* < 0.01; Unifrac test), demonstrating that unforeseen environmental factors did not influence the development of surface communities.

### 2.6 The Artificial Biofilm Changes the Phylogenetic Structure of Natural Communities

**Figure 5:**
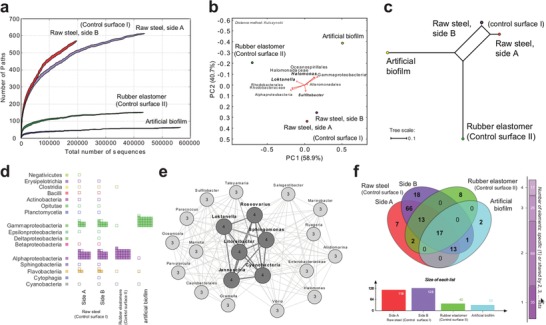
Comparison of natural bacterial communities that developed on the modified surfaces exposed to seawater in the Gulf of Naples, Italy. a) Rarefaction curves describing the α‐diversity. b) PcoA analysis using the Kulczynski β‐diversity matrix. Biplot of PC1 and PC2, representing 99.6% variability, shows sample observations and first 10 variability vectors. c) Neighbor‐Net analysis showing the similarity between the analyzed samples. Scale represents distance. d) Phylogenetic structure over all four samples analyzed. Class is used as rank. Squares designate presence and quantity of each taxon. e) Venn diagram demonstrating the intersections of samples by showing the number of shared taxa between samples. f) The core biome of the analyzed samples as calculated by Megan6. Threshold (%): 0.001, Min./Max. Prevalence (%): 75/100, Probability (%): 75. Numbers designate the number of samples where the taxon was observed. Dark thick lines connect taxa detected in all four samples.

**Figure 6:**
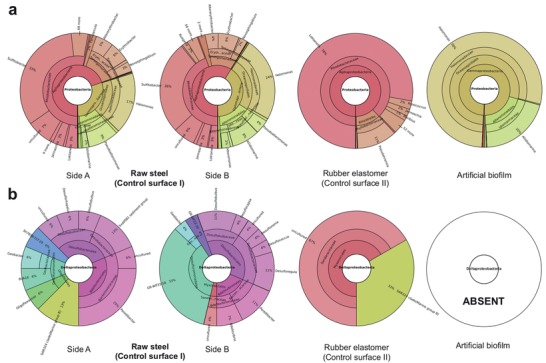
Composition of phylum Proteobacteria and class δ‐Proteobacteria as assessed by SILVAngs. a) General overview of the most abundant phylum Proteobacteria. b) Overview of class δ‐proteobacteria, which includes common sulphate‐reducing bacteria. See Supporting information for an interactive representation of community structure in each sample.

In the second field experiment, we successfully obtained the structure of the developed natural bacterial communities using molecular biology tools. Based on the16S rRNA gene we identified the metagenetic information describing the composition of each community that had developed on the test surfaces exposed in the environment for 42 DOE. The 16S rRNA gene metagenetic sequencing confirmed that the structure of bacterial communities differed between different types of the exposed surface (**Figure** **5**). Both of the protective coatings, the artificial biofilm solution and the rubber elastomer (control surface II) showed lower community diversity compared to the untreated raw steel surface (control surface I) (Table S3, Supporting Information), with the artificial biofilm exhibiting the lowest number of assigned taxonomic units (Figure 5a). The α‐ and γ‐proteobacteria were the dominant groups in all samples tested (**Figures** 5d and **6**). Compared to the raw steel (control surface I), the artificial biofilm caused the δ‐proteobacteria, particularly *Halomonas* sp. (78%) and *Aliidiomarina* (20%), to quantitatively predominate in the community, unlike for the rubber elastomer (control surface II), which selected out the α‐proteobacteria instead, particularly genus *Loktanella* (78%) and *Pseudahrensia* (11%) (Figure 6). Additionally, dominant groups present on raw steel surface, like *Sulfitobacter* (30%) were reduced by both surface treatments, although more by the artificial biofilm, which reduced it down to 0.01%, compared to the 0.4% of the rubber elastomer (control surface II) (Figure 6; multimedia material S3, Supporting Information). Furthermore, the δ‐proteobacteria were completely absent in the artificial biofilm sample, while some sequences from this group, mostly identified as uncultured representatives, were identified in the surface of the rubber elastomer (control surface II) (Figure 6a). The *Bacillus* sp. taxon was detected in very low amounts in the artificial biofilm sample, however, the presence of DEV1 strain was not detected using this approach.

The core biome analysis demonstrated that the network of observed taxa, was divided into two parts, depending on the type of protective coating used, with the set of taxa including *Loknanella, Cyanobacteria, Litoreibacter*, and *Sphingomonas* that were present in all tested samples (Figure 5e). The qualitative intersection between the tested samples demonstrated that the majority of the detected taxa present in either the artificial biofilm or the rubber elastomer (control surface II) were shared with the control raw steel samples (control surface I) and around half of the taxa were also shared between these two samples (Figure 5f). In absolute terms the dominant groups represented more than 90% of all sequences and the unique phylogenetic taxa were represented by 2% of the sequences, when the coatings (artificial biofilm or control rubber elastomer) were compared to control raw steel surface. On the functional level, the differences in composition translated to a lower metabolic potentials of the bacterial communities in both, the artificial biofilm and the control rubber elastomer samples (Figure S4 and Text S4, Supporting Information). The artificial biofilm, in particular, mainly caused an absence of several metabolic pathways in the community, like the pathways for the degradation of biopolymers, aerobic respiration, H_2_ and sufide oxidation, nitrate and sulfate reduction and the mevalonate pathway among others. On the other hand, the potential was increased for only a few pathways, H_2_ reduction being affected the most.

## 3 Discussion

Our study demonstrates the proof of concept for a new approach against material corrosion, where by constructing artificial bacterial biofilms on the surface of steel, we interfere with the natural process of microbial colonization to exclude MIC‐causing bacteria. The artificial biofilm can affect the biofouling process immediately after the material is exposed in the environment, in time range of seconds to minutes, as it is known that the formation of conditioning films and the attachment of the first bacterial colonizers from the environment occurs in this time frame (reviewed in^37^). According to our results, the constructed artificial biofilm does not inhibit the colonization process per se, but rather causes a structural change of the newly forming natural microbial surface community. The observed effect may be the result of the presence of bacterial cells composing the artificial biofilm, the deposited polyelectrolytes, particularly polysulfonated lignin and chitosan, the right combination of all these components or also the physical properties of the modified surface as a whole.

The applied bacterial cells can act in three different ways. First, by exposing receptors on the cell surface and interacting with the cells colonizing the surface from the environment, they promote the attachment of a limited set of bacterial species.^19^ Second, they form new local niches with specific physicochemical properties, such as high or low pH, absence of oxygen, and low nutrient content.^19^ Third, they interfere with bacterial growth by producing antimicrobial compounds, such as antibiotics and bacteriocins, or by interfering with quorum sensing.^38^, ^39^ Both of the strains selected for this study (*B. pumilus* and *B. brevis*) can produce antimicrobial compounds that decrease the growth of different gram negative and gram positive bacteria.^40^, ^41^ Additionally, the inhibition of the colonization of the surface by new microbes can be contact‐dependent, as was attributed to many strains of *Bacillus*.^42^, ^43^ To have the highest impact of the selected bacteria, we included the two types of encapsulated cells in different parts of our artificial biofilm. First, we encapsulated the gramicidin S producing cells of strain DSM30 with artificial PEs, i.e., PEI and PAA, and deposited them in the bottom part (Table S1, Supporting Information) in order to ensure antimicrobial protection in case of cracking or damaging of the rubber elastomer water‐resistant barrier. Since the gramicidin S was already produced during culturing and was enclosed within the PE nanocapsules, to be released in case the elastomer barrier was compromised, the survival of these cells was not needed. Furthermore, gramicidin S primarily did not effect the changes in the upper part of the biofilm (growth of stain DEV1 and all the colonizing strains) and only came into effect if the lower part of the biofilm was exposed to seawater. Second, we chose to use viable antimicrobial‐producing cells of environmental strain DEV1 to fill up the empty spaces on the surface of the rubber elastomer, which are targeted by microbial colonizers from the environment. To improve their deposition to the surface by increasing their “stickiness,” we encapsulated them with natural PEs chitosan and lignosulphonate. It is to be noted that as long as the initial perturbation of the normal biofouling process was strong enough to change the course of natural biofilm development, long‐term survival of the applied cells was not necessary. Indeed, 16S rRNA gene sequencing could not confirm the presence of strain DEV1, indicating that the natural community initially influenced by the artificial biofilm finally did not include the deposited bacterial cells of strain DEV1 or their levels were too low to be detected by our analytical methods.

The polymeric constituents of the artificial biofilm as well as the polymers formed by the cells composing the artificial biofilm can make a strong selection of the microorganisms that will colonize the surface.^44^ In our solution the natural PEs were primarily in direct contact with the environment, whereas the artificial PEs would only be so, if the bottom part of the biofilm got exposed to seawater. Natural polymers like lignosulphonates and chitosan are biomimetic,^45^ act as antimicrobials^46^, ^47^ and also lower application costs, since they are industrial waste.^48^ Lignin itself has similar bacteriostatic and biocidal activity,^49^ is a very abundant and is mostly unexploited. Alternatively, seawater is a source of chitosan degrading bacteria, meaning that chitosan in the artificial will attract these bacteria, possibly causing the degradation of the original biofilm structure. The representatives from the genera *Halomonas* and *Aliidiomarina* have been reported to have chitosan degrading abilities^50^ and according to our results, they were abundant on the surfaces coated by the artificial biofilm (Figure 5). Since such an establishment of a bacterial ecological group can further select the colonizers from the environment, careful selection of the PEs will reinforce the functionality and value of the final artificial biofilm solution.

The third important component of the artificial biofilm was the rubber elastomer. Its application was necessary to establish protection against the fast chemical corrosion, producing iron oxides and increased surface roughness. We were expecting that the absence of such protection would lead to increased volume of the corroded layer, formation of cavities and the tearing of the prepared artificial biofilm structure. The exposed raw corroded surface would allow free colonization of the surface by microorganisms from the environment and would diminish the effect of the applied components of the artificial biofilm. A comparison of the control surfaces used in our study (raw metal—control surface I and rubber elastomer—control surface II) shows that the physical water‐resistant elastomer barrier already notably contributes to the changes of the biomass of the microorganisms on the surface (Table S2, Supporting Information), but it selects out different bacteria than does the full artificial biofilm solution (Figures 4, 5, and 6). The fact that the rubber elastomer alone (control surface II) selects out taxa like *Loktanella* (Figure 6) and other representatives of the Roseobacter group, which has been related to algal blooms^51^ and sulfur cycling^52^ indicates that the physical barrier alone does not prevent MIC‐related bacteria to populate and dominate the surface.

In the described artificial biofilm solution we tried to incorporate as many of the different factors that influence the establishment of bacteria that colonize the freshly exposed surfaces, in order to increase the probability of perturbing the development of natural biofilms. As demonstrated by our field experiments in seawater the artificial biofilm affects the initial stages of surface colonization by microbes from the environment (Figure 4), which in turn determines the later composition of the community (Figures 5 and 6). The artificial biofilm selects for γ‐proteobacteria, particularly bacteria from the genus *Halomonas*, which are known to produce large amounts of exopolysaccharides^53^ inhibiting the settlement of invertebrate larvae.^54^ The artificial biofilm also completely prevents the establishment of corrosion‐related δ‐proteobacteria, which include sulfate reducing bacteria^1^ and Fe reducers *Geobacter* and *Schewanella*,^55^ that were present in the control surfaces (Figure 6). On the functional level, the artificial biofilm clearly reduced the general metabolic potential of the community (Figure S4 and Text S2, Supporting Information). In relation to natural biofilm formation it reduced the degradation of natural extracellular biopolymers, which can contribute to the effects of the selected PEs described above, to modulate the structure of the developing natural biofilm.^56^ In relation to the progression of the corrosion process, it reduced the communities' potential for aerobic respiration and hydrogen, as well as sulfite oxidation. At the same time, the reduced sulfur and nitrate reduction processes alongside the increased H_2_ production demonstrate a reduced production of corrosion causing compounds (H_2_S) and a reduced potential for the oxidation of Fe.

More generally, we have shown with this study that the surface of different *Bacillus* strains can be electrostatically modified using artificial (Table 1) or natural polyelectrolytes (Table 2). Previous studies have already shown that the LBL approach can be applied also to gram negative bacteria,^57^ meaning that the artificial biofilm approach allows the deposition of different types of bacteria as mono‐ or heterogenic multilayered consortia. Moreover, our approach enables the attachment of microorganisms that naturally have problems attaching to specific surfaces, are not capable of forming biofilms and are not adapted to the micro‐environments on the surface. Since the LBL approach helps overcome these problems, the cells do not need to directly interact with the surface nor be viable on the long run, as long as they influence or interfere with the development of natural biofilms. The possibility to deposit bacterial cells directly to the metal surface (Figure 2) or the surface of the metal‐protecting elastomer coating (Figure 3) demonstrates that the LBL approach can be used to construct artificial biofilms on surfaces of different materials once they are themselves electrostatically appropriately modified (Table 3). This shows a wide‐reaching impact on the development of biobased materials. The concept of this study is thus applicable to different fields, like material science, biotechnology, medicine, and eco‐remediation, but it opens up several questions that need further examination. Future studies need to address: i) how the artificial biofilms evolve or collapse on inert surfaces, ii) how the deposited bacteria multiply and spread on the surface or how they detach from it, iii) how different bacterial strains or their combinations effect the colonization by single preselected bacteria from the environment, and iv) how important is the PE matrix for the attachment of colonizing bacteria or for exhibiting antimicrobial activity alongside the antibacterial strains used to construct the biofilm.

We conclude that artificially constructed biofilms are a promising ecological and evolutionary approach to protect the surface of materials as well as to help us study and understand the general principles of of biofilm development.

## 4 Experimental Section

*Bacterial Strains*: Two bacterial strains were used for the construction of artificial biofilms, type strain *B. brevis* DSM 30 (DSMZ, Germany) and marine environmental isolate DEV1 (Inbiotec, Spain), which shares 99.4% similarities in 16S rRNA gene sequence with the type strain *B. pumilus* ATCC 7061 (gen. bank accession no. AY876289) (see Text S1, Supporting Information). Cells of *B. brevis* were producing antibiotic gramicidin S,^58^ while for *B. pumilus* strain DEV1, antimicrobial activity against bacteria *Bacillus subtilis*, *Micrococcus luteus*, *Escherichia coli*, and *Desulfovibrio vulgaris* has been demonstrated (Inbiotec, Spain; personal communication). Both bacterial cultures were cultured on standard nutrient medium No. 1 (Sigma, USA) at 30 °C under aerobic growth conditions and were stored at −80 °C for long‐term storage.

*Electrostatic Modification of the Cell Surface*: The surfaces of bacterial cells were modified by depositing PEs onto the surface of the cell in consecutive layers (Figure 1). The deposition of each PE layer was monitored by the electrophoretic light scattering (ELS) measurements (see below). To test the deposition protocol, the cell surface of *B. brevis* strain DSM30 was modified by the artificial PEs, positively charged polyethyleneimine (PEI[+]) and either the negatively charged PSS[−] or polyacrylic acid (PAA[−]), while the cell surface of strain DEV1 was modified by natural PEs, the positively charged chitosan[+] and the negatively charged lignosulfonate[−]. For the final biofilm formulation, PAA was selected instead of PSS, due to its lower resistance to biological degradation (reviewed in 36]. The procedure for LBL modification of the cell surface was carried out as follows. Both bacterial strains were grown in No. 1 nutrient broth medium (Sigma‐Aldrich, USA) at 30 °C and 250 rpm using an orbital shaker incubator (Neolab, Germany) overnight untill they reached the late exponential to early stationary phase (OD_600_ = 1–1.4). The cells of *B. brevis* were collected by centrifugation (20 000 × *g*) and were washed once with 10 × 10^−3^ m NaH_2_PO_4_, pH = 7.4 (PBS) and twice with 0.2 × 10^−3^ m PBS. The pellet was finally resuspended in 1/20 of original volume using 0.2 × 10^−3^ m PBS. Residual aggregates were broken down by a 15 min vortex mixing (Vortex 3, IKA, Germany). The first PE was deposited onto the cell surface by combining equal volumes of the prepared cell suspension and 1% (w/v) PEI[+] (*M*_w_ 600 000–1 000 000; Sigma‐Aldrich, USA), pH 7.4, vortexing the mixture for 15 min, collecting the cells by centrifugation at 3000 × *g*, washing them three times in 0.2 × 10^−3^ m PBS, resuspending them again in 1/20 of original volume using 0.2 × 10^−3^ m PBS and finally vortexing the suspension for 15 min to break down the residual aggregates. To deposit the second layer, the procedure was repeated with 1% (w/v) PSS[−] (*M*_w_ ≈ 70 000; Sigma‐Aldrich, USA) or 1% (w/v) PAA[−] (*M*_w_ ≈ 100 000; Sigma‐Aldrich, USA). To deposit the third layer the cell suspension was again combined with an equal volume of 1% (w/v) PEI[+], pH 7.4, then this mixture was used to deposit the cells onto the modified metal surface during construction of the artificial biofilm (see below). The modification of the cell surface of strain DEV1 was carried out using: PBS adjusted to 5.6, 0.1% (w/v) chitosan[+] (Sigma‐Aldrich, USA), pH 5.6, and 1% (w/v) lignosulfonate[−] (*M*_w_ ≈ 8000, *M*_n_ ≈ 3000; Sigma‐Aldrich, USA), pH, 5.6. As before, three layers of PEs were prepared and the cells, resuspended in chitosan, were deposited to the modified metal surface when preparing the artificial biofilm (see below).

*Electrostatic Modification of the Metal Surface and Deposition of Cells*: The LBL modification of the metal surface was carried out by the dip coating approach, where the PEs were applied by immersing the metal panel in the respective PE coating solutions. Standard steel panel, *W* × *L* × *T* = 100 × 150 × 0.81 mm (Q‐panel R‐46, Q‐Lab Corp., USA) and standard stainless steel panel, *W* × *L* × *T* = 76 × 152 × 0.89 mm (Q‐panel SS‐36, Q‐Lab, Germany) (Figure S3, Supporting Information) were used to test and visualize each consecutive step of surface modification. To protect it from the fast chemical corrosion and formation of Fe‐oxides on the surface, the standard steel panel was cleaned with 1 m NaOH, sterile water, and acetone and coated it with a primer (Plasti Dip Grundierung Spray, Plasti Dip Grundierung GmBH, Germany) and a nonchlorinated rubber elastomer (Plasti Dip Flüssiggummi Spray, Plasti Dip Grundierung GmBH, Germany). After this step, the LBL modification of the surface was started by immersing the metal panel in 1% (w/v) solution of alumina nanowires[+] (alumina; size 2–6 nm x 200–400 nm; Sigma‐Aldrich, USA), pH 7.6, for 30 min, followed by 1% (w/v) solution of lignosulphonate[−], pH 7.6 for 30 min, 1% PEI[+] solution with the resuspended pre‐encapsulated cells of strain DEV1 (see above) for 30 min and again 1% (w/v) solution of lignosulphonate[−] for 30 min. Each step, i.e., the deposition of a single layer, was followed by gentle rinsing the surface with miliQ water. The deposition of each layer was monitored by ELS and SEM (see below).

*ELS Measurements*: The electrostatic properties of surfaces were measured by ELS with the DelsaNano HC system (Beckman Coulter, USA). Mobilities of the cells or standard charged particles were used as indicators of the charges of the un‐/modified cell or metal surfaces, respectively, and served as the basis for the calculation of the surface ζ‐potential. The ζ‐potential was used as a measure to infer the charge of the modified surface (negative versus positive).

For the ELS measurements of the un‐/modified bacterial cells, the cells were washed 5 times with 10 × 10^−3^ m PBS, pH 7.4, and were finally diluted 30 times in 0.2 × 10^−3^ m PBS, pH 7.4 (OD_600_ << 0.1). The analysis was performed using the Standard Flow Cell (Beckman Coulter, USA) and the Delsa Nano software (Beckman Coulter, USA), using the following parameters: 25˚C, 60 V fixed voltage, Smoluchowski conversion and linear autocorrelation function. The Aqueous Suspension of Standard Polystyrene Latex (Conc. 1%, −70.3 mV ± 10%; Otsuka Electronics, Japan) was used as the standard. The ELS measurements of the un‐/modified metal surface were carried out using metal coupons (R‐46; Q‐Lab Corp., USA), size 3 cm^2^, Standard Particles for Flat Surface Cell (Beckman Coulter, USA) and the Flat Surface Flow Cell (Beckman Coulter, USA). The modified surface of steel coupons was washed with miliQ water. The standard particles were diluted 250 times in miliQ water and the solution was used to measure the surface ζ‐potential. The analysis was performed with the Delsa Nano software (Beckman Coulter, USA), using the parameters as described above.

*SEM and Image Analysis*: The metal surface was visualized using ultra high resolution FE‐SEM based on the GEMINI Technology (SUPRA 35 VP, Carl Zeiss, Germany). Metal coupons (R‐46 and SS‐36; Q‐Lab Corp., USA), size 1 cm^2^ were used without prior preprocessing or contrasting. The surface roughness and size of the particles were assessed by image analysis using ImageJ (Fiji). Image sections, one fifth of the viewing field, were taken to obtain 25 independent measurements. Surface roughness was defined as the coefficient of variation of gray values, which were the result of scattering of electrons on the surface towards the fixed angle detector. Particle size was assessed using the default ImageJ Analyze particles macro, with the default image Threshold setting and a particle size cutoff of 10 pixels. All statistical analysis between datasets describing the physical properties were performed using the Mann–Whitney nonparametric test.

*Construction of the Multilayered Artificial Biofilm*: The full artificial biofilm solution was constructed on the surface of standard steel (R‐46) and consisted of two sections, i) the bottom part composed of strain DSM30 cells and ii) the upper part composed of strain DEV1 cells, separated by a protective layer of Plasti Dip rubber (Table S1, Supporting Information). The bottom part of the biofilm, was constructed on top of the Plasti Dip adhesion primer, which was applied directly to the steel surface. The following layers composed the bottom part: alumina[+] and lignosulphonate[−], modified cells[+] of strain DSM30, alumina[+], lignosulphonate[−] and again modified cells[+] of strain DSM30. After air‐drying, the bottom part was enclosed within a water‐tight layer of Plasti Dip rubber elastomer[−]. The top part of the biofilm above the rubber elastomer layer, was constructed from 3 layers of modified cells[+] of strain DEV1 (see above), each time separated by a layer of chitosan[+] and a layer of lignosulfonate[−]. The panels were stored in chitosan suspension containing modified cells[+] of strain DEV1 during transport to the field testing sites in Napoli, Italy and Piran, Slovenia. The unmodified R‐46 steel panel and the R‐46 steel panel coated only by the Plasti Dip primer and rubber elastomer were used as controls.

*Environmental Field Test*: To assess the temporal development of microbial communities the first field testing was carried out on the Adriatic coast in Piran, Slovenia (45°31'03.3”N 13°34'05.9”E), where the test panels were submerged in seawater for 28 days, March through April, and were monitored every 7 days. To assess the phylogenetic structure of the developed surface community the second field testing was performed in an area with naturally elevated levels of sulfates^59^ on the Mediterranean coast in the Gulf of Naples, Italy (40°48'29.5”N 14°09'34.2”E), where the test panels were submerged in seawater for a total of 42 days, March through April (Figure S3, Supporting Information). The samples were transferred to the environmental test sites in sealed sterile bags (SteriBag, Bürkle, Germany). After the exposure period, the plates were washed by dipping the panels into fresh seawater 3 times, to remove all macroscopic particles that were loosely attached to the surface. The panels were stored and transferred in sterile plastic bags (SteriBag; Bürkle, Germany) to the laboratory, where they were stored at 4 °C before DNA isolation.

*DNA Isolation*: The surface associated natural bacterial communities (natural biofilms) were collected aseptically by thorough scraping of the surface and subsequently wiping the surface using sterile FLOQSwabs swabs (Copan Flock Technologies, Italy). DNA extraction was performed according to^60^ and the purified DNA was stored at −20 °C prior to further analysis.

*DGGE Analysis*: The temporal profiling of the natural bacterial surface communities was analyzed by DGGE as described in.^60^ To assess the similarities between different treatments of the metal surface, a Unifrac analysis of the tree was carried out using UniFrac from the QIIME software package.^61^

*16S rRNA Gene Sequencing*: For 16S rRNA metagenetic sequencing, the purified DNA was fluorescently quantified using SybrGreen I fluorescent nucleic acid stain (ThermoFisher, USA), DNA quantification standards (Invitrogen, USA) and SynergyH4 multiplate reader (Biotek, USA). The purity was assessed by checking the absorption ratio at 260/280 nm using the Take3 plate (Biotek, USA) on the SynergyH4 system. To obtain sufficient amounts of highly pure intact DNA, the samples were pooled together (Table S1, Supporting Information) to produce two control samples (sides A and B) of the raw steel surface (control surface I), one control sample of the rubber‐elastomer‐coated steel (control surface II) and one sample of the full artificial biofilm solution (sample surface). Next‐generation sequencing was carried out using the 16S Microbiome profiling pipeline (Eurofins, Germany) based on the V1‐V3 variable regions of the 16S rRNA gene.

*Bioinformatic Analysis*: Read QC filtering was performed using local and web‐based tools available from the Galaxy project,^62^ read identification was performed using Blast+^63^ in combination and RDP database v11_5.^64^ Comparison of community structures, diversity indices and core meta‐genome was assessed using MEGAN6.^65^ Venn diagram was constructed using the web platform MetaCoMET using the standard MEGAN6 output.^66^ The α‐diversity and read identification on the genus level was additionally assessed by the SILVAngs pipeline 1.3.9^67^ and the interactive representation of the phylogenetic structure was visualized using Krona v2.7.^68^ The metabolic inference was calculated on a random subset of 10 000 16S rRNA sequences from each sample using the PAthway PRediction by phylogenetIC plAcement (PAPRICA) v0.4.1b.^69^ When noted, statistical comparisons between data sets were done using student's T test.

## Conflict of Interest

The authors declare no conflict of interest.

## Supporting information

Supplementary
