# Supplementary material for: Natural Microbial Communities Can Be Manipulated by Artificially Constructed Biofilms
Source: Adv Sci (Weinh). 2019 Sep 19;6(22):1901408. doi: 10.1002/advs.201901408 (PMC6865284; doi:10.1002/advs.201901408)
Supplement: Supplementary file 1 — Supplementary [file ADVS-6-1901408-s001.pdf]

Copyright WILEY-VCH Verlag GmbH & Co. KGaA, 69469 Weinheim, Germany, 2019.

ADVANCED SCIENCE
Open Access

# Supporting Information

for *Adv. Sci.,* DOI: 10.1002/advs.201901408

## Natural Microbial Communities Can Be Manipulated by Artificially Constructed Biofilms

*Tomaž Rijavec, Jan Zrimec, Robvan Spanning, and Aleš Lapanje**

# Supporting Information

## Natural microbial communities can be manipulated by artificially constructed biofilms

Tomaž Rijavec[1,2], Jan Zrimec[3,2], Rob van Spanning[4], Aleš Lapanje[1,2]

[1] Department of Environmental Sciences, Jožef Stefan Institute, Jamova cesta 39, 1000 Ljubljana, Slovenia

[2] Institute of Metagenomics and Microbial Technologies, Clevelandska ulica 19, 1000 Ljubljana, Slovenia

[3] Systems and Synthetic Biology, Chalmers University of Technology, Kemivägen 10, 412 96 Göteborg, Sweden

[4] Systems Bioinformatics, Faculty of Science, Vrije Universiteit Amsterdam, De Boelelaan 1105, 1081 HV Amsterdam, The Netherlands

# SUPPORTING INFORMATION: TABLES

**Table S1.** A summary of the three types of surfaces that were exposed to seawater in natural environment. Raw steel surface (Control surface I) and steel protected by the rubber elastomer coating (Control surface II) were used as control surfaces to be compared against the sample surface of the artificial biofilm solution. Due to the low concentration of DNA obtained from the surfaces of test coupons after the incubation experiment, the material collected from both of the sides of the test coupon (sides A and B) was pooled together for each sample as indicated in the table. The final formulation of the artificial biofilm consisted of two layers of *B. brevis* DSM30 cells, covered hermetically sealed by a layer of liquid rubber, followed by three layers of cells of environmental strain DEV1.

| | *Type* | *Sample* | *Schematic representation* | *Exposed Coupon* | *Sample labels* | *No. sequences* |
|---|---|---|---|---|---|---|
| Tested samples | *Control surface I* | **Raw Steel** | 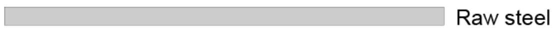  | Side A | Label: **STEEL-A** | 415974 |
| | | | | Side B | Label: **STEEL-B** | 189312 |
| | *Control surface II* | **Rubber elastomer** | 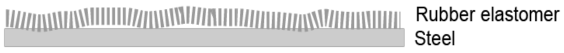  | Side A<br>Side B | Label: **RUBBER** (Pooled sample) | 416773 |
| | *Sample surface* | **Artificial Biofilm Solution** | 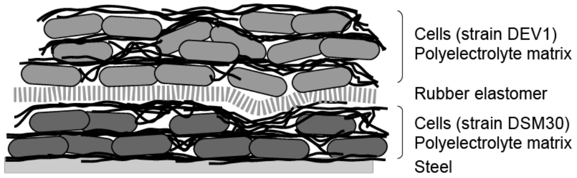  | Side A<br>Side B | Label: **BIOFILM** (Pooled sample) | 540208 |

**Table S2.** Indirect assessment of microbial biomass on surface using molecular quantification of microbial DNA. DNA was quantified at different times of surface exposure to the environment to assess the biomass of the developing natural microbial biofilms. Each side (A, B) of the experimental plate was examined separately. Data is expressed as ng $cm^{-2}$.

| | Raw Steel (Control Surface I) | | Rubber Elastomer Coating (Control Surface II) | | Artificial biofilm solution | |
|---|---|---|---|---|---|---|
| **Exposure time** | **Side A** | **Side B** | **Side A** | **Side B** | **Side A** | **Side B** |
| 7 day[a] | <0[BD] | <0[BD] | <0[BD] | <0[BD] | <0[BD] | <0[BD] |
| 14 day[a] | <0[BD] | <0[BD] | 1.3 | 2.8 | <0[BD] | 2.6 |
| 28 day[a] | <0[BD] | <0[BD] | 3.4 | 2.6 | 2.6 | 3.6 |
| 42 day[b] | 13.9 | 17.1 | 0.13 | <0[BD] | 0.1 | <0[BD] |

[a] Experimental location: coastline of Adriatic sea, Piran, Slovenia

[b] Experimental location: coastline of Mediterranean Sea, Napoli, Italy

**Table S3.** Diversity indices as calculated by MEGAN6 using leaves on the taxonomy tree. Genus used as the rank for calculations.

| **Sample** | | **Shannon – Weaver Index** | **Simpson – Reciprocal Index** |
|---|---|---|---|
| Raw steel (Control Surface I) | *Side A* | 3.169 | 4.648 |
| | *Side B* | 3.338 | 5.405 |
| Rubber elastomer (Control surface II) | *Pooled* | 1.029 | 1.407 |
| Artificial biofilm solution | *Pooled* | 0.909 | 1.553 |

# SUPPORTING INFORMATION: FIGURES

**Fig. S1.** Attachment of the positively charged electrostatically modified bacterial cells to the negatively charged surfaces of glass and rubber elastomer protected steel. The surfaces of cells were modified by electrostatic deposition of 3 layers, PEI[+], PAA[-] and PEI[+]), and the modified cells were deposited to the raw negatively charged glass surface (a, c) and the negatively charged surface of rubber elastomer covering steel (b, d). Images a-d visualize the cells attached to the surface after the surface was exposed to the cell suspension with high (c, d) and low concentrations (a, b); $OD_{600}$ values were > 1.0 and < 0.8, respectively. The surface of the glass coupons was examined by differential interference contrast (DIC) microscopy and the surface of the rubber elastomer by fluorescence microscopy, both using the AxioObserver Z1 system (Zeiss, Germany). Fluorescence stain Syto82 (Invitrogen, USA) was used to stain viable cells to obtain a fluorescence signal. Scale bar is indicated as white line. The % of the surface area covered by cells and number of cells per surface area (e, f) were assessed by image analysis using ImageJ (Fiji). Image sections, one fifth of the viewing field, were take to obtain 25 independent measurements of the presented surface area. Student's t-test was used to measure statistically significant difference between data sets. P value: $p < 0.001$ (***) and $p < 0.01$ (**). Incubation of the samples in suspension of modified cells for 7 days, showed that after extensive rinsing, the cells were not washed off and were still attached to the surface of the test coupons (*data not shown*).

**Fig. S2.** Visualization of the first four layers of artificial biofilm on the surface of stainless steel using the LBL approach. (a,b) Raw unmodified surface of steel. (c, d) Deposit of the first positively charged

layer of Alumina[+]. (e,f) Deposit of the second negatively charged layer of lignosulphonate[-] on top of the first layer of Alumina. (g,h) Deposit of the third positively charged layer of encapsulated cells[+] covering the layer of lignosulphonate[-] and Alumina[+]. (i, j) Deposit of the fourth negatively charged layer of lignosulphonate[-] covering the first three layers.

**Fig. S3.** Preparation of the artificial biofilm and field testing experiment. (a) Standard steel (R-46) and stainless steel (SS-36) panels (Q-Lab, Germany) used for the experiments. Large panels are cut down to small coupon size to fit Desla Nano flow cell and SEM chamber for determination of surface ζ-potential and visualization of the surface, respectively. (b) Coating of standard steel (R-46) only by rubber elastomer protective coating to be used as control (Control surface II). (c) Preparation of the artificial biofilm is carried out by immersing the metal panels in polyelectrolytes or suspensions of LBL encapsulated cells. Here, the deposition of a layer of Alumina nanowires[+] (*left*) and lignosulphonate[-] (*right*) is presented. (d) The deposition of the bacterial cells is carried out by immersing the panel in the suspension of the positively charged encapsulated cells[+]. The suspension is also used for storage and transport of the panels to the environmental test site. (e) The test locations on the coast of the Adriatic sea in Piran, Slovenia and the coast of the Mediterranean sea in the Gulf of Napoly, Italy. (f) The panels are mounted onto the lift and submerged into the seawater; location shown, Piran, Slovenia. (g) The samples are collected aseptically and stored in sterile bags prior to processing and isolation of DNA from the surface; here, samples after 42 DOE in seawater in the Gulf of Naples, Italy are presented.

**Fig. S4.** The metabolic profiles of the surface bacterial communities are changed do to the application of the artificail biofilm or the control rubber elastomer coating. Data represents factors of change

(increase or decrease) for the range of 879 metabolic processes which are calculated by comparing each of the two samples implementing a protective coating (artificial biofilm or rubber elastomer) to the averaged sample with the raw surface of steel (average of samples A and B). The metabolic potential is reduced in both samples compared to the raw steel sample, but the effect of each surface treatment ( artificial biofilm or rubber elastomer) is functionally different as demonstrated by the difference in metabolic changes in each sample. See also Text S4 in SI for a detailed description.

**Fig. S5.** Demonstration of the thickness of the deposit of 2 layers, Alumina[+] and lignosulphonate[-], on the surface of (a, b) rubber elastomer coating covering the R-46 steel and (c, d) SS-36 stainless steel. A scratch, removing the deposit, is made as reference, to demonstrate the thickness of the deposit.

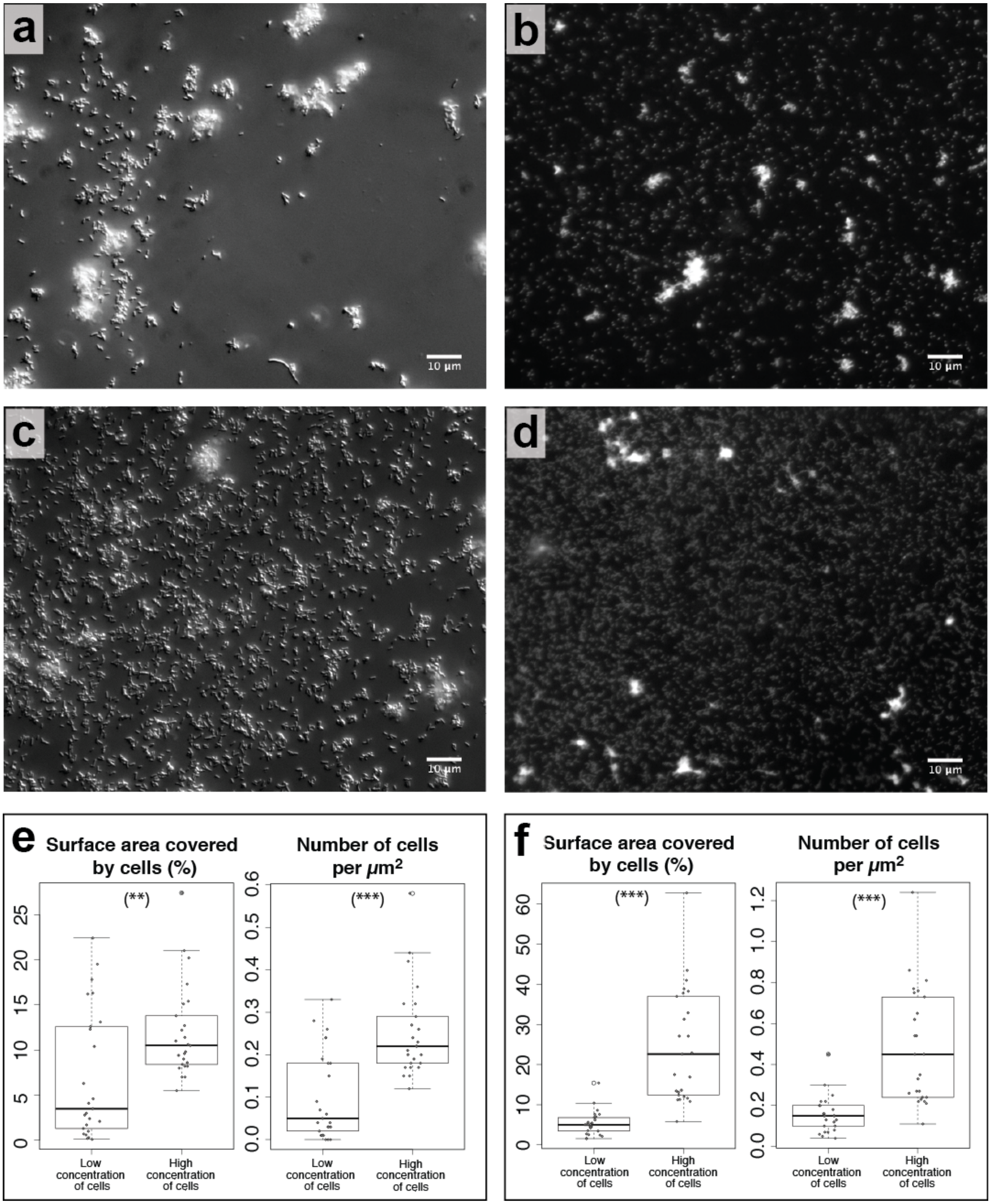
a
b
c
d
10 µm
e
Surface area covered by cells (%)
Number of cells per $\mu m^2$
(**)
(***)
Low concentration of cells
High concentration of cells
f
Surface area covered by cells (%)
Number of cells per $\mu m^2$
(***)
(***)
Low concentration of cells
High concentration of cells

Fig. S1

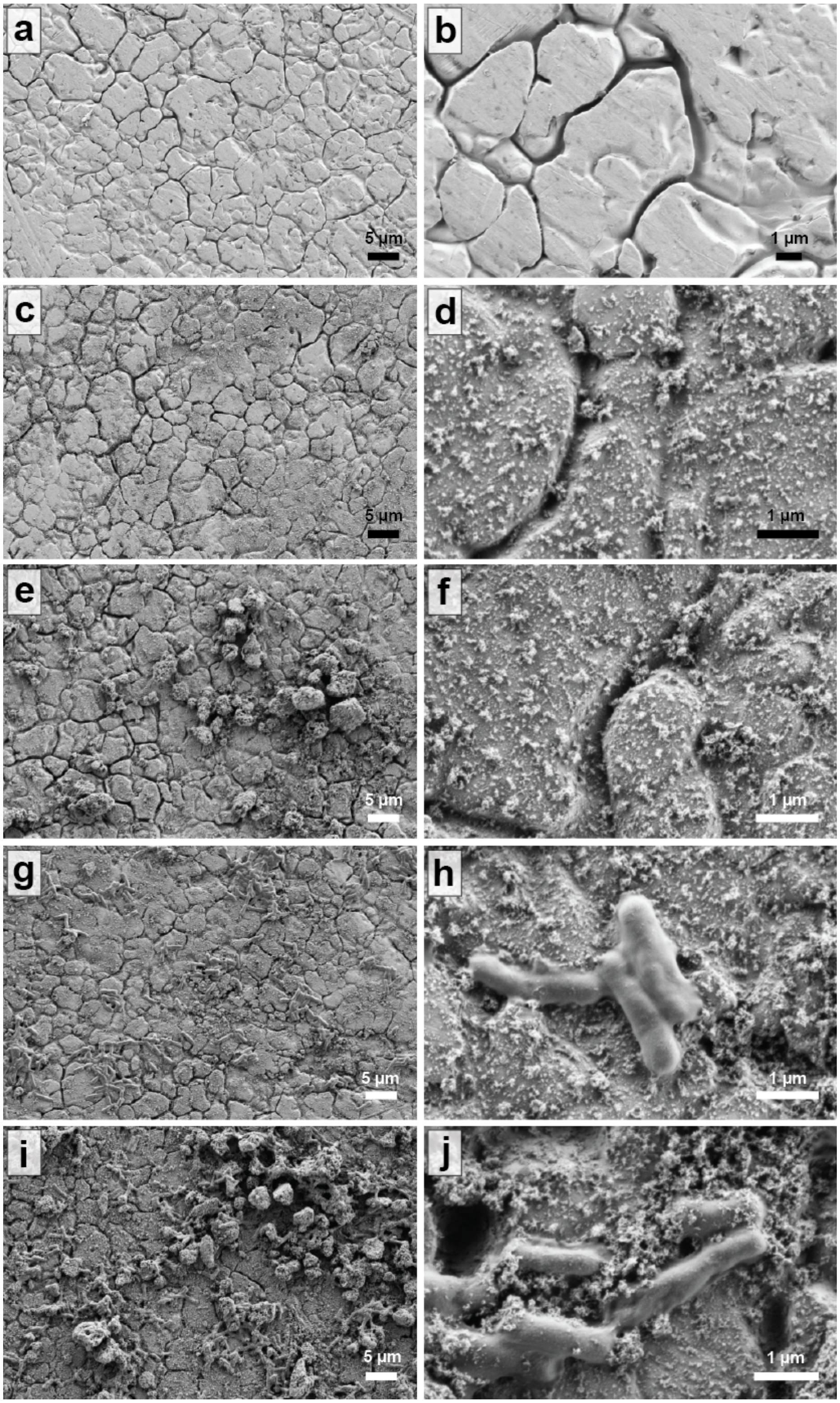


Fig. S2

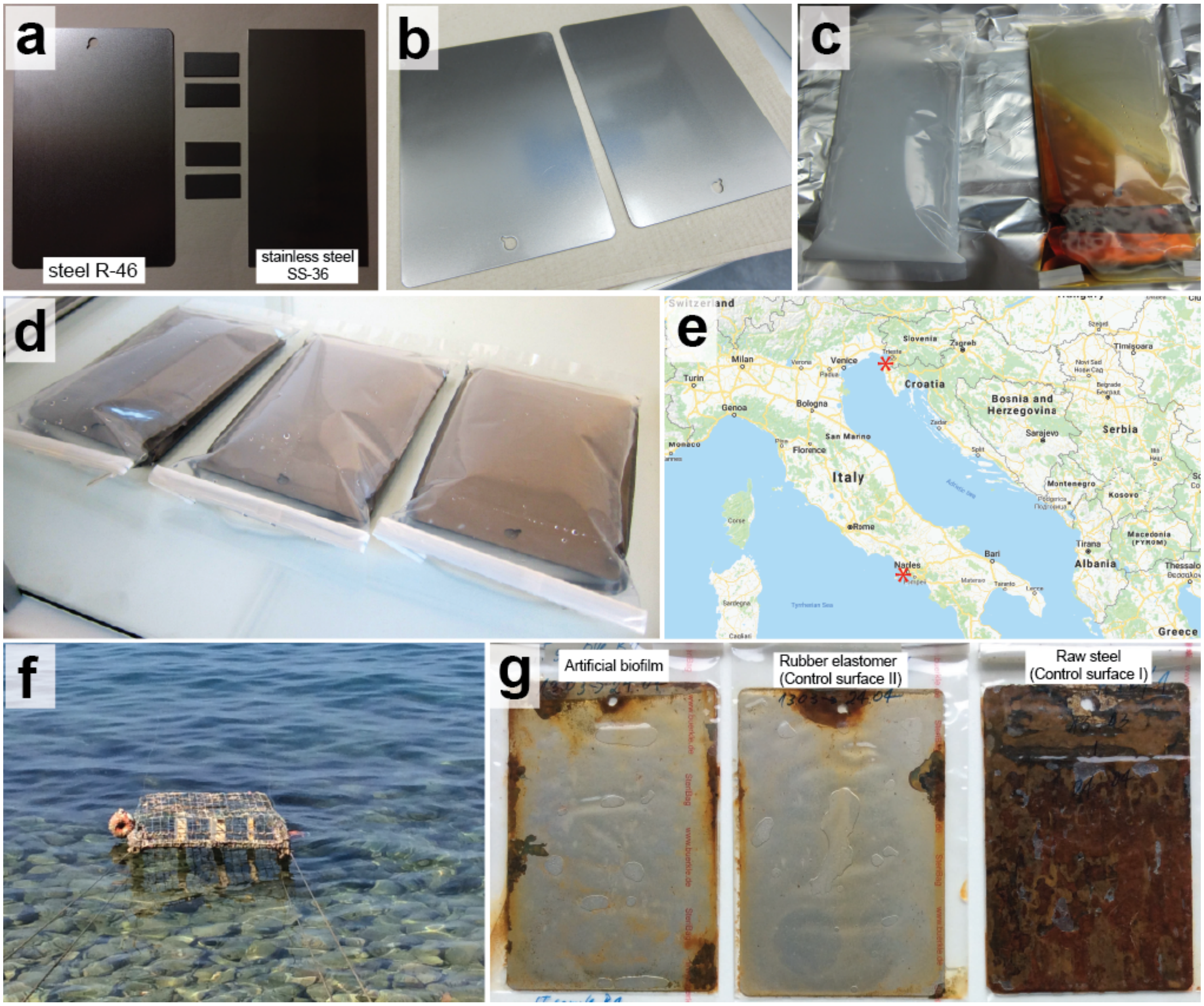
a
steel R-46
stainless steel
SS-36
b
c
d
e
f
g
Artificial biofilm
Rubber elastomer
(Control surface II)
Raw steel
(Control surface I)

Fig. S3

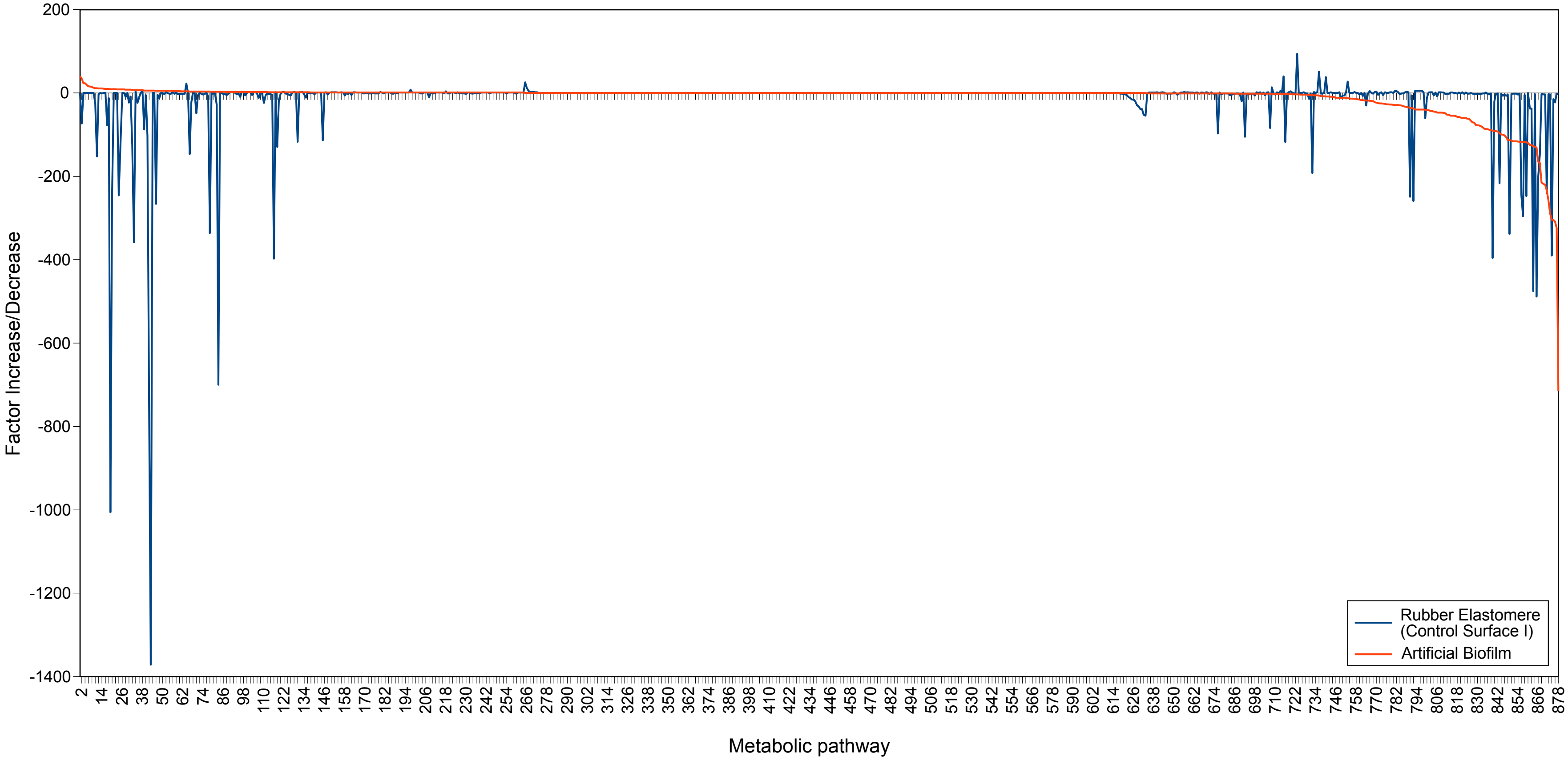
200
0
-200
-400
-600
-800
-1000
-1200
-1400
Factor Increase/Decrease
Metabolic pathway
Rubber Elastomere (Control Surface I)
Artificial Biofilm
2
14
26
38
50
62
74
86
98
110
122
134
146
158
170
182
194
206
218
230
242
254
266
278
290
302
314
326
338
350
362
374
386
398
410
422
434
446
458
470
482
494
506
518
530
542
554
566
578
590
602
614
626
638
650
662
674
686
698
710
722
734
746
758
770
782
794
806
818
830
842
854
866
878

Fig. S4

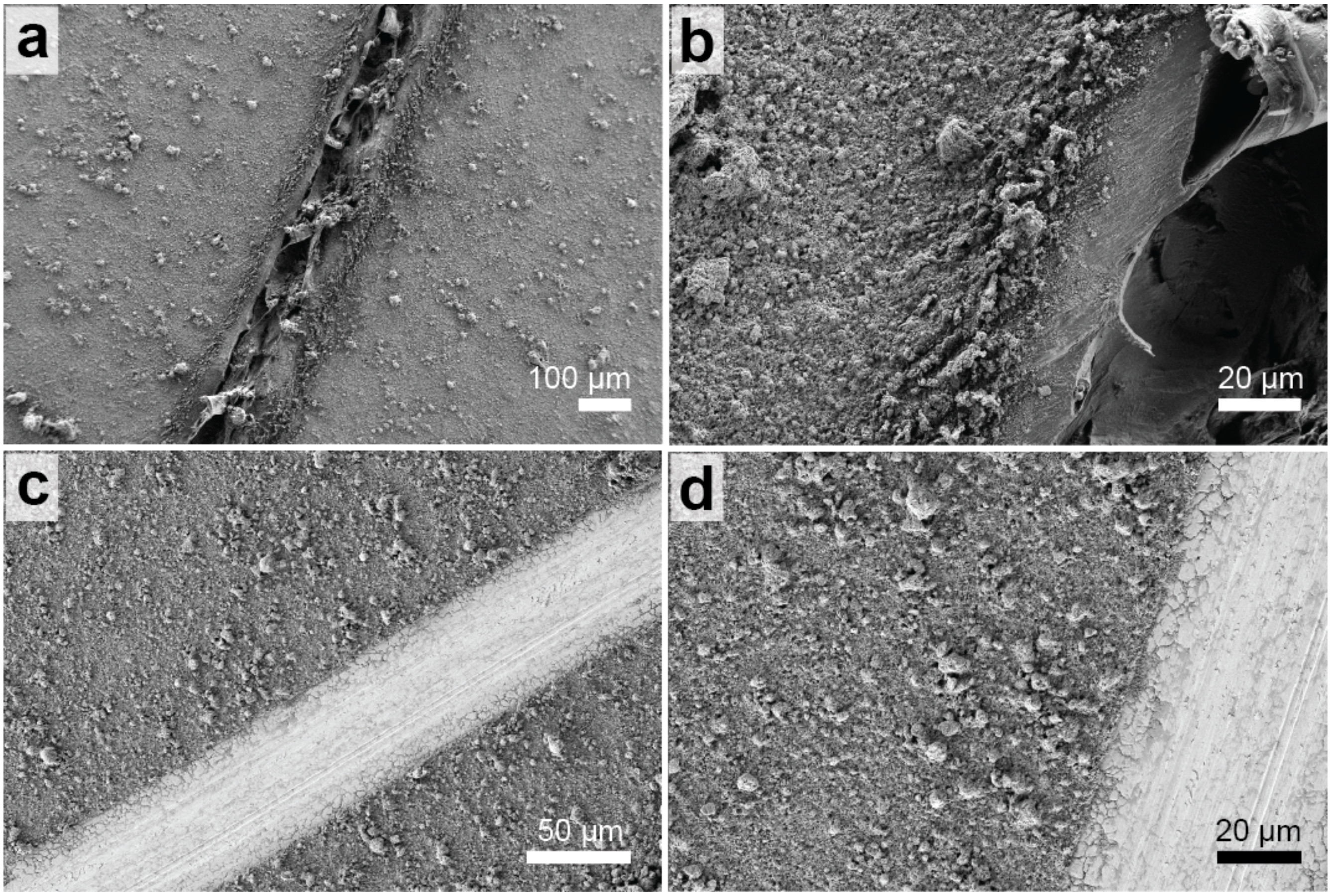


Fig. S5

# SUPPORTING INFORMATION: MULTIMEDIA MATERIAL S1

*see* Krona .html output available as compressed .zip file.

# SUPPORTING INFORMATION: TEXT S1

**Text S1.** Identification of the marine environmental isolate DEV1 is based on sequencing of the 16S rRNA gene. Sequence identity was determined using the Seqmatch program (version 3; RDP project) and the RDP database (Release 11, Update 5, Sept. 30, 2016).

>DEV1_seq16SrRNA

TCGGAGAGTTTGATCCTGGCTCAGGACGAACGCTGGCGGCGTGCCTAATACATGCAAGTCG

AGCGGACAGAAGGGAGCTTGCTCCCGGATGTTAGCGGCGGACGGGTGAGTAACACGTGGG

TAACCTGCCTGTAAGACTGGGATAACTCCGGGAAACCGGAGCTAATACCGGATAGTTCCTT

GAACCGCATGGTTCAAGGATGAAAGACGGTTTCGGCTGTCACTTACAGATGGACCCGCGGC

GCATTAGCTAGTTGGTGGGGTAATGGCTCACCAAGGCAACGATGCGTAGCCGACCTGAGAG

GGTGATCGGCCACACTGGGACTGAGACACGGCCCAGACTCCTACGGGAGGCAGCAGTAGG

GAATCTTCCGCAATGGACGAAAGTCTGACGGAGCAACGCCGCGTGAGTGATGAAGGTTTT

CGGATCGTAAAGCTCTGTTGTTAGGGAAGAACAAGTGCGAGAGTAACTGCTCGCACCTTGA

CGGTACCTAACCAGAAAGCCACGGCTAACTACGTGCCAGCAGCCGCGGTAATACGTAGGTG

GCAAGCGTTGTCCGGAATTATTGGGCGTAAAGGGCTCGCAGGCGGTTTCTTAAGTCTGATG

TGAAAGCCCCCGGCTCAACCGGGGAGGGTCATTGGAAACTGGGAAACTTGAGTGCAGAAG

AGGAGAGTGGAATTCCACGTGTAGCGGTGAAATGCGTAGAGATGTGGAGGAACACCAGTG

GCGAAGGCGACTCTCTGGTCTGTAACTGACGCTGAGGAGCGAAAGCGTGGGGAGCGAACA

GGATTAGATACCCTGGTAGTCCACGCCGTAAACGATGAGTGCTAAGTGTTAGGGGGTTTCCG

CCCCTTAGTGCTGCAGCTAACGCATTAAGCACTCCGCCTGGGGAGTACGGTCGCAAGACTG

AAACTCAAAGGAATTGACGGGGGCCCGCACAAGCGGTGGAGCATGTGGTTTAATTCGAAG

CAACGCGAAGAACCTTACCAGGTCTTGACATCCTCTGACAACCCTAGAGATAGGGCTTTCC

CTTCGGGGACAGAGTGACAGGTGGTGCATGGTTGTCGTCAGCTCGTGTCGTGAGATGTTGG

GTTAAGTCCCGCAACGAGCGCAACCCTTGATCTTAGTTGCCAGCATTTAGTTGGGCACTCTA

AGGTGACTGCCGGTGACAAACCGGAGGAAGGTGGGGATGACGTCAAATCATCATGCCCCT

TATGACCTGGGCTACACACGTGCTACAATGGACAGAACAAAGGGCTGCGAGACCGCAAGG

TTTAGCCAATCCCATAAATCTGTTCTCAGTTCGGATCGCAGTCTGCAACTCGACTGCGTGAA

GCTGGAATCGCTAGTAATCGCGGATCAGCATGCCGCGGTGAATACGTTCCCGGGCCTTGTAC

ACACCGCCCGTCACACCACGAGAGTTTGCAACACCCGAAGTCGGTGAGGTAACCTTTATGG

AGCCAGCCGCCGAAGGTGGGGCAGATGATTGGGGTGAAGTCGTAACAAGGTAGCCGTATC

GGAAGGTGCGGCTGGATCACCTCCTTTCTA

## A. TYPE STRAIN SEQUENCE MATCH

| | |
|---|---|
| Seqmatch: | version 3 |
| RDP Data: | release11_5 |
| Data Set: | both type and non-type strains, both environmental (uncultured) sequences and isolates, near-full-length sequences (>1200 bases), good quality sequences |
| Comments: | 12758 sequences were included in the search<br>The screening was based on 7-base oligomers |
| Query Submit Date: | Thu May 24 03:11:59 EDT 2018 |
| Match hit format: | short ID, S_ab score, unique common oligomers and sequence full name. More help is available. |
| S_ab score: | The S_ab score is percentage of shared 7-mers between two sequences, which does not require the alignment for calculation |
| number nt: | Unique common oligomers |

Lineage:
Results for Query Sequence: seqmatch_seq, 1456 unique oligos

rootrank Root (20) (match sequences)
domain Bacteria (20)
phylum Firmicutes (20)
class Bacilli (20)
order Bacillales (20)
family Bacillaceae 1 (20)
genus Bacillus (20)

| | | | |
|---|---|---|---|
| S000003473 | 0.888 | 1423 | Bacillus subtilis (T); DSM10; AJ276351 |
| S000006847 | 0.888 | 1435 | Bacillus mojavensis (T); IFO15718; AB021191 |
| S000012241 | 0.892 | 1440 | Bacillus vallismortis (T); DSM11031; AB021198 |
| S000014133 | 0.898 | 1426 | Bacillus atrophaeus (T); JCM9070; AB021181 |
| S000417318 | 0.960 | 1415 | Bacillus altitudinis (T); type strain:41KF2b; AJ831842 |
| S000428475 | 0.890 | 1337 | Bacillus subtilis (T); NRRL B-23049; AF074970 |
| S000458519 | 0.984 | 1354 | Bacillus safensis (T); FO-036b; AF234854 |
| S000481068 | 0.994 | 1352 | Bacillus pumilus (T); ATCC 7061; AY876289 |
| S001014161 | 0.969 | 1443 | Bacillus stratosphericus (T); type strain:41KF2a; AJ831841 |
| S001014162 | 0.969 | 1443 | Bacillus aerophilus (T); type strain:28K; AJ831844 |
| S002234727 | 0.886 | 1374 | Bacillus tequilensis (T); 10b; HQ223107 |
| S002287782 | 0.887 | 1456 | Bacillus amyloliquefaciens (T); FZB42; CP000560 |
| S002288068 | 0.895 | 1453 | Bacillus amyloliquefaciens (T); FZB42; CP000560 |
| S002290003 | 0.889 | 1453 | Bacillus amyloliquefaciens (T); FZB42; CP000560 |
| S002290710 | 0.886 | 1452 | Bacillus amyloliquefaciens (T); FZB42; CP000560 |
| S002291243 | 0.886 | 1453 | Bacillus amyloliquefaciens (T); FZB42; CP000560 |
| S003257857 | 0.888 | 1446 | Bacillus subtilis (T); type strain: DSM 22148; HE582781 |
| S004007309 | 0.968 | 1426 | Bacillus xiamenensis (T); MCCC 1A00008; JX680066 |
| S004065189 | 0.886 | 1452 | Bacillus amyloliquefaciens (T); FZB42; CP000560 |
| S004071803 | 0.983 | 1338 | Bacillus invictae (T); Bi.FFUP1; JX183147 |

## B. TYPE AND NON-TYPE STRAIN SEQUENCE MATCH

| | |
|---|---|
| Seqmatch: | version 3 |
| RDP Data: | release11_5 |
| Data Set: | both type and non-type strains, both environmental (uncultured) sequences and isolates, near-full-length sequences (>1200 bases), good quality sequences |
| Comments: | 1558793 sequences were included in the search<br>The screening was based on 7-base oligomers |
| Query Submit Date: | Tue Feb 06 09:48:05 EST 2018 |
| Match hit format: | short ID, orientation, similarity score, S_ab score, unique common oligomers and sequence full name. More help is available. |
| S_ab score: | The S_ab score is percentage of shared 7-mers between two sequences, which does not require the alignment for calculation |
| number nt: | Unique common oligomers |

Lineage:
Results for Query Sequence: seqmatch_seq, 1456 unique oligos

rootrank Root (20) (match sequences)
domain Bacteria (20)
phylum Firmicutes (20)
class Bacilli (20)
order Bacillales (20)
family Bacillaceae 1 (20)
genus Bacillus (20)

| | | | |
|---|---|---|---|
| S000446614 | 0.996 | 1416 | Bacillus sp. 19499; LMG 19499; AJ315067 |
| S001155226 | 0.996 | 1422 | Bacillus pumilus; CTSP17; EU855198 |
| S001155227 | 0.996 | 1422 | Bacillus pumilus; CTSP18; EU855199 |
| S001155229 | 0.996 | 1422 | Bacillus pumilus; CTSP20; EU855201 |
| S001155242 | 0.996 | 1422 | Bacillus pumilus; CTSP37; EU855214 |
| S001155243 | 0.996 | 1422 | Bacillus pumilus; CTSP38; EU855215 |
| S001155246 | 0.996 | 1422 | Bacillus pumilus; CTSP44; EU855218 |
| S004007317 | 0.996 | 1424 | Bacillus pumilus; MCCC 1A00439; JX680074 |
| S004007349 | 0.996 | 1424 | Bacillus pumilus; MCCC 1A06991; JX680106 |
| S004007350 | 0.996 | 1424 | Bacillus pumilus; MCCC 1A06996; JX680107 |
| S004007351 | 0.996 | 1424 | Bacillus pumilus; MCCC 1A07053; JX680108 |
| S004007353 | 0.996 | 1424 | Bacillus pumilus; MCCC 1A07286; JX680110 |
| S004007372 | 0.996 | 1424 | Bacillus pumilus; MCCC 1A08151; JX680129 |
| S004007373 | 0.996 | 1424 | Bacillus pumilus; MCCC 1A08152; JX680130 |
| S004007374 | 0.996 | 1424 | Bacillus pumilus; MCCC 1A08153; JX680131 |
| S004007375 | 0.996 | 1424 | Bacillus pumilus; MCCC 1A08154; JX680132 |
| S004092011 | 0.997 | 1431 | Bacillus pumilus; H4.9/8; KJ722435 |
| S004226722 | 0.997 | 1296 | marine bacterium IVA004; KJ814556 |
| S004255267 | 0.996 | 1424 | Bacillus pumilus; MCCC1A06453; KC346445 |
| S004474570 | 0.998 | 1232 | Bacillus pumilus; 46_5_il; KP699764 |

# SUPPORTING INFORMATION: TEXT S2

**Text S2.** Representation of the metabolic steps that are increased/reduced in the bacterial communities affected by the two protective coatings (the artificial biofilm and the control rubber elastomer) compared to the average profile obtained from the communities that had developed in raw surface of steel. The metabolic profiles are prepared with PAPRICA v0.4.1b (Bowman and Ducklow, 2015) using a subset of 10000 sequences prepared by bioawk (https://github.com/lh3/bioawk). The data is represented as factor increase/decrease of abundance compared to the raw steel sample and is ordered by size according to the artifiial biofilm sample.

Reference: J. S. Bowman,H. W. Ducklow, *PLoS One*, **2015**, *10(8)*, e0135868.

| | Control surface I (Rubber elastomer) | Artificial biofilm |
|---|---|---|
| hydrogen production V | 0.000 | 40.206 |
| anthranilate degradation I (aerobic) | -73.188 | 34.120 |
| L-arginine degradation II (AST pathway) | 0.000 | 23.383 |
| S-methyl-5'-thioadenosine degradation IV | 0.000 | 23.279 |
| citrate lyase activation | 0.000 | 19.296 |
| putrescine degradation II | 0.000 | 15.815 |
| glycolate and glyoxylate degradation I | 0.000 | 15.564 |
| NAD salvage pathway II | 0.000 | 14.488 |
| trehalose degradation I (low osmolarity) | 0.000 | 12.412 |
| tetrathionate reduction I (to thiosulfate) | -25.811 | 11.231 |
| benzene degradation | -152.533 | 11.187 |
| L-arabinose degradation I | -3.031 | 10.798 |
| autoinducer AI-2 biosynthesis I | 0.000 | 10.674 |
| thio-molybdenum cofactor biosynthesis | -1.188 | 10.622 |
| pectin degradation II | 0.000 | 10.141 |
| mannitol degradation I | 0.000 | 9.845 |
| fructose degradation | -77.386 | 9.788 |
| gellan degradation | -12.353 | 9.405 |
| L-alanine biosynthesis II | -1006.099 | 9.262 |
| mevalonate degradation | -240.864 | 8.870 |
| demethylmenaquinol-6 biosynthesis I | 0.000 | 8.862 |
| demethylmenaquinol-9 biosynthesis | 0.000 | 8.862 |
| D-sorbitol degradation II | 0.000 | 8.744 |
| tetrapyrrole biosynthesis I (from glutamate) | -245.660 | 8.721 |
| resorcinol degradation | -129.378 | 8.493 |
| trans-3-hydroxy-L-proline degradation | 0.000 | 8.433 |
| Kdo transfer to lipid IVA II | 0.000 | 8.398 |
| spermidine biosynthesis III | -9.351 | 7.940 |
| ADP-L-glycero-beta-D-manno-heptose biosynthesis | 0.000 | 7.742 |
| ethanolamine utilization | -23.139 | 7.722 |
| L-arginine degradation III (arginine decarboxylase/agmatinase pathway) | -8.316 | 7.700 |
| octaprenyl diphosphate biosynthesis | -126.576 | 7.374 |
| 8-amino-7-oxononanoate biosynthesis I | -358.415 | 7.128 |
| L-valine degradation II | 4.097 | 7.002 |
| L-cysteine degradation II | -23.832 | 6.839 |
| cytidylyl molybdenum cofactor biosynthesis | -10.115 | 6.735 |
| L-threonine degradation I | 0.000 | 6.630 |
| D-threitol degradation | 6.763 | 6.589 |
| S-methyl-5-thio-alpha-D-ribose 1-phosphate degradation | -87.782 | 5.792 |
| reductive monocarboxylic acid cycle | -5.270 | 5.665 |
| L-histidine degradation I | -103.666 | 5.619 |
| 2-amino-3-carboxymuconate semialdehyde degradation to 2-oxopentenoate | -752.124 | 5.607 |
| 5-(carboxymethoxy)uridine biosynthesis | -1371.937 | 5.596 |
| ribose degradation | 0.000 | 5.580 |
| L-ascorbate degradation I (bacterial. anaerobic) | 0.000 | 5.380 |
| tetrahydromonapterin biosynthesis | -266.019 | 5.354 |
| S-methyl-5'-thioadenosine degradation I | -4.497 | 5.326 |
| arginine dependent acid resistance | -12.207 | 5.280 |
| D-galactarate degradation I | 0.000 | 5.100 |
| D-glucarate degradation I | 0.000 | 5.092 |

| | Control surface I (Rubber elastomer) | Artificial biofilm |
|---|---|---|
| L-glutamate degradation II | -2.745 | 5.035 |
| D-gluconate degradation | 1.574 | 4.903 |
| nitrate reduction VIII (dissimilatory) | 0.000 | 4.901 |
| L-tryptophan degradation to 2-amino-3-carboxymuconate semialdehyde | -2.529 | 4.858 |
| thiamine formation from pyrithiamine and oxythiamine (yeast) | -1.907 | 4.842 |
| 2-aminoethylphosphonate degradation I | 3.560 | 4.527 |
| D-arginine degradation | -2.448 | 4.475 |
| ectoine biosynthesis | 1.327 | 4.441 |
| L-threonine degradation IV | -2.757 | 4.374 |
| ethanol degradation I | -3.189 | 4.293 |
| chlorosalicylate degradation | -2.076 | 4.187 |
| methylsalicylate degradation | -2.076 | 4.187 |
| salicylate degradation I | -2.076 | 4.187 |
| benzoyl-CoA degradation III (anaerobic) | 22.514 | 4.040 |
| L-phenylalanine biosynthesis II | 0.000 | 3.935 |
| demethylmenaquinol-8 biosynthesis I | -146.913 | 3.621 |
| CMP-pseudaminate biosynthesis | -22.049 | 3.562 |
| thiazole biosynthesis II (aerobic bacteria) | 1.454 | 3.495 |
| D-serine degradation | 0.000 | 3.491 |
| 2-O-alpha-mannosyl-D-glycerate degradation | -48.679 | 3.456 |
| creatinine degradation II | -5.280 | 3.424 |
| chitobiose degradation | 0.000 | 3.302 |
| lipoate biosynthesis and incorporation II | -2.522 | 3.271 |
| ethanol degradation II | -3.909 | 3.269 |
| lipoate salvage I | -2.524 | 3.268 |
| sulfoacetaldehyde degradation III | 0.000 | 3.221 |
| cholesterol degradation to androstenedione I (cholesterol oxidase) | -8.147 | 3.210 |
| beta-alanine biosynthesis III | -336.029 | 3.198 |
| myo-inositol degradation I | 2.442 | 3.143 |
| L-malate degradation II | -2.203 | 3.075 |
| 4-deoxy-L-threo-hex-4-enopyranuronate degradation | 1.327 | 3.032 |
| glutathionylspermidine biosynthesis | -28.256 | 2.889 |
| 4-nitrotoluene degradation II | -700.218 | 2.826 |
| D-arabinose degradation I | 0.000 | 2.771 |
| fucose degradation | 0.000 | 2.771 |
| base-degraded thiamine salvage | -2.900 | 2.687 |
| thiamine salvage IV (yeast) | -2.900 | 2.687 |
| CDP-diacylglycerol biosynthesis II | -5.024 | 2.582 |
| thiosulfate disproportionation III (rhodanese) | -2.213 | 2.577 |
| L-selenocysteine biosynthesis I (bacteria) | 1.967 | 2.573 |
| adenosine nucleotides degradation II | 3.009 | 2.570 |
| 2.4.6-trichlorophenol degradation | 2.152 | 2.531 |
| L-lactaldehyde degradation (anaerobic) | 0.000 | 2.517 |
| D-glucarate degradation II | -3.541 | 2.515 |
| fatty acid beta-oxidation III (unsaturated. odd number) | -1.085 | 2.463 |
| acyl carrier protein metabolism | -9.750 | 2.432 |
| purine ribonucleosides degradation | 3.553 | 2.399 |
| 4-aminobutanoate degradation III | 1.610 | 2.377 |
| mixed acid fermentation | -5.437 | 2.306 |
| cardiolipin biosynthesis I | 0.000 | 2.304 |
| putrescine biosynthesis I | 1.987 | 2.262 |

| | Control surface I (Rubber elastomer) | Artificial biofilm |
|---|---|---|
| N-acetylglucosamine degradation I | 1.756 | 2.222 |
| 6-hydroxymethyl-dihydropterin diphosphate biosynthesis I | -4.979 | 2.214 |
| adenine and adenosine salvage III | 2.101 | 2.212 |
| peptidoglycan maturation (meso-diaminopimelate containing) | 2.186 | 2.191 |
| pyrimidine deoxyribonucleotides de novo biosynthesis II | -1.929 | 2.157 |
| NAD salvage pathway IV | -11.894 | 2.151 |
| guanine and guanosine salvage II | 1.815 | 2.130 |
| 2-methylcitrate cycle I | -1.076 | 2.096 |
| thiamine diphosphate biosynthesis II (Bacillus) | -24.134 | 2.033 |
| mannosylglycerate biosynthesis I | -5.658 | 1.992 |
| L-tyrosine degradation I | -1.345 | 1.963 |
| phenylacetate degradation I (aerobic) | -2.784 | 1.921 |
| D-galactose degradation I (Leloir pathway) | -2.911 | 1.869 |
| sulfate reduction III (assimilatory) | -4.573 | 1.848 |
| pyrimidine ribonucleosides salvage I | -397.644 | 1.848 |
| thiazole biosynthesis I (facultative anaerobic bacteria) | 1.614 | 1.827 |
| chitin degradation II | -129.962 | 1.737 |
| chlorinated phenols degradation | -16.302 | 1.737 |
| pyrimidine deoxyribonucleotides de novo biosynthesis I | -2.566 | 1.701 |
| TCA cycle II (plants and fungi) | 2.306 | 1.669 |
| D-fructuronate degradation | 2.338 | 1.647 |
| 4-amino-2-methyl-5-diphosphomethylpyrimidine biosynthesis | -1.772 | 1.644 |
| 4-aminobutanoate degradation II | -2.220 | 1.637 |
| NAD biosynthesis I (from aspartate) | -4.259 | 1.596 |
| preQ0 biosynthesis | -6.483 | 1.595 |
| pyrimidine deoxyribonucleosides degradation | 1.308 | 1.572 |
| inosine-5'-phosphate biosynthesis II | 1.982 | 1.559 |
| dTDP-L-rhamnose biosynthesis I | 1.082 | 1.556 |
| 1.4-dihydroxy-2-naphthoate biosynthesis | -117.149 | 1.546 |
| acetate formation from acetyl-CoA I | 1.394 | 1.530 |
| guanine and guanosine salvage | 1.523 | 1.517 |
| creatinine degradation I | 2.073 | 1.514 |
| proline to cytochrome bo oxidase electron transfer | -3.741 | 1.483 |
| spermidine biosynthesis I | -11.629 | 1.451 |
| gondoate biosynthesis (anaerobic) | 1.652 | 1.399 |
| polyhydroxybutanoate biosynthesis | -1.139 | 1.392 |
| 4-hydroxybenzoate biosynthesis V | 0.000 | 1.377 |
| pyrimidine nucleobases salvage I | 1.368 | 1.371 |
| succinate to cytochrome bd oxidase electron transfer | -3.346 | 1.345 |
| adenosine deoxyribonucleotides de novo biosynthesis II | 1.005 | 1.344 |
| guanosine deoxyribonucleotides de novo biosynthesis II | 1.005 | 1.344 |
| glycolysis III (from glucose) | 1.370 | 1.336 |
| lipid IVA biosynthesis | 1.083 | 1.331 |
| biotin biosynthesis from 8-amino-7-oxononanoate I | -113.728 | 1.328 |
| pentose phosphate pathway (non-oxidative branch) | 1.058 | 1.322 |
| allantoin degradation to ureidoglycolate II (ammonia producing) | 1.696 | 1.308 |
| succinate to cytochrome bo oxidase electron transfer | -4.146 | 1.286 |
| UTP and CTP dephosphorylation I | 1.058 | 1.280 |
| L-glutamate biosynthesis I | 1.835 | 1.268 |
| L-glutamine degradation II | 1.835 | 1.268 |
| heme biosynthesis I (aerobic) | 1.858 | 1.268 |

| | Control surface I (Rubber elastomer) | Artificial biofilm |
|---|---|---|
| L-asparagine degradation I | 1.528 | 1.262 |
| flavin biosynthesis I (bacteria and plants) | 1.314 | 1.260 |
| CDP-diacylglycerol biosynthesis III | 1.133 | 1.256 |
| 2.3-dihydroxybenzoate biosynthesis | 1.454 | 1.249 |
| xanthine and xanthosine salvage | 1.360 | 1.243 |
| glycerophosphodiester degradation | -6.167 | 1.234 |
| choline degradation III | 0.000 | 1.229 |
| acyl-CoA hydrolysis | -2.632 | 1.205 |
| L-glutamine biosynthesis I | 1.206 | 1.204 |
| phosphatidylethanolamine biosynthesis I | -5.272 | 1.201 |
| thymine degradation | 1.626 | 1.191 |
| phosphatidylcholine biosynthesis VI | 1.604 | 1.187 |
| selenate reduction | 1.094 | 1.182 |
| palmitoleate biosynthesis I (from (5Z)-dodec-5-enoate) | 1.150 | 1.177 |
| UDP-alpha-D-glucuronate biosynthesis (from UDP-glucose) | 1.144 | 1.173 |
| L-glutamate degradation I | 1.065 | 1.167 |
| Entner-Doudoroff pathway I | -1.047 | 1.163 |
| L-proline degradation | -1.089 | 1.150 |
| methylglyoxal degradation I | 1.100 | 1.144 |
| 4-hydroxyphenylpyruvate biosynthesis | -1.074 | 1.144 |
| L-phenylalanine biosynthesis I | -1.074 | 1.144 |
| cis-vaccenate biosynthesis | -1.073 | 1.140 |
| 2'-deoxy-alpha-D-ribose 1-phosphate degradation | 1.234 | 1.135 |
| trans. trans-farnesyl diphosphate biosynthesis | -1.177 | 1.134 |
| geranyl diphosphate biosynthesis | -1.179 | 1.127 |
| ppGpp biosynthesis | -1.072 | 1.125 |
| benzoate degradation II (aerobic and anaerobic) | 2.164 | 1.124 |
| guanosine nucleotides degradation III | 1.241 | 1.115 |
| L-tryptophan degradation I (via anthranilate) | -1.058 | 1.112 |
| L-tyrosine biosynthesis I | 1.249 | 1.107 |
| ubiquinol-8 biosynthesis (prokaryotic) | 1.106 | 1.104 |
| purine deoxyribonucleosides degradation I | 1.202 | 1.103 |
| L-citrulline degradation | 1.101 | 1.102 |
| ubiquinol-7 biosynthesis (prokaryotic) | -1.568 | 1.102 |
| ubiquinol-9 biosynthesis (prokaryotic) | -1.568 | 1.102 |
| UDP-glucose biosynthesis | 1.217 | 1.089 |
| glutathione-glutaredoxin redox reactions | -1.108 | 1.088 |
| glycerol degradation I | 1.074 | 1.084 |
| glyoxylate cycle | 1.082 | 1.081 |
| L-glutamate biosynthesis III | 1.067 | 1.078 |
| L-serine degradation | 1.076 | 1.072 |
| TCA cycle I (prokaryotic) | 1.145 | 1.069 |
| tRNA processing | 1.065 | 1.064 |
| myo-inositol biosynthesis | 1.064 | 1.063 |
| N-acetylneuraminate and N-acetylmannosamine degradation I | 7.758 | 1.060 |
| L-proline biosynthesis I | 1.061 | 1.060 |
| L-leucine degradation I | 1.045 | 1.060 |
| long-chain fatty acid activation | 1.057 | 1.057 |
| protocatechuate degradation II (ortho-cleavage pathway) | 1.308 | 1.053 |
| molybdenum cofactor biosynthesis | 1.055 | 1.053 |
| phosphopantothenate biosynthesis I | -1.194 | 1.052 |

| | Control surface I (Rubber elastomer) | Artificial biofilm |
|---|---|---|
| glycolysis I (from glucose 6-phosphate) | -1.004 | 1.047 |
| glutathione biosynthesis | 1.045 | 1.044 |
| L-ornithine biosynthesis I | 1.045 | 1.043 |
| L-homocysteine biosynthesis | 1.018 | 1.040 |
| UDP-N-acetyl-alpha-D-mannosaminouronate biosynthesis | -10.736 | 1.040 |
| fatty acid beta-oxidation I | 1.025 | 1.037 |
| adenosine deoxyribonucleotides de novo biosynthesis | -1.161 | 1.036 |
| guanosine deoxyribonucleotides de novo biosynthesis I | -1.161 | 1.036 |
| guanylyl molybdenum cofactor biosynthesis | 1.318 | 1.036 |
| L-alanine biosynthesis III | 1.072 | 1.032 |
| L-lysine biosynthesis I | 1.031 | 1.031 |
| urate biosynthesis/inosine 5'-phosphate degradation | 1.213 | 1.030 |
| [2Fe-2S] iron-sulfur cluster biosynthesis | 1.073 | 1.029 |
| L-alanine degradation IV | -1.185 | 1.029 |
| benzoyl-CoA degradation I (aerobic) | 3.890 | 1.028 |
| 5-aminoimidazole ribonucleotide biosynthesis II | -1.171 | 1.027 |
| 4-aminobenzoate biosynthesis | -1.216 | 1.025 |
| L-isoleucine biosynthesis I (from threonine) | 1.020 | 1.020 |
| L-valine biosynthesis | 1.019 | 1.018 |
| L-leucine biosynthesis | 1.018 | 1.017 |
| glutaminyl-tRNAgln biosynthesis via transamidation | -1.165 | 1.016 |
| hydroxymethylpyrimidine salvage | 1.722 | 1.016 |
| L-cysteine biosynthesis I | -1.187 | 1.014 |
| L-histidine biosynthesis | 1.015 | 1.013 |
| pyridoxal 5'-phosphate biosynthesis I | -1.188 | 1.012 |
| UDP-N-acetyl-D-glucosamine biosynthesis I | -1.189 | 1.010 |
| 4-aminobutanoate degradation I | -2.166 | 1.010 |
| palmitate biosynthesis II (bacteria and plants) | 1.007 | 1.009 |
| stearate biosynthesis II (bacteria and plants) | 1.008 | 1.009 |
| tetrahydrofolate salvage from 5.10-methenyltetrahydrofolate | -1.193 | 1.009 |
| N6-L-threonylcarbamoyladenosine37-modified tRNA biosynthesis | -1.868 | 1.008 |
| adenosylcobalamin salvage from cobalamin | 1.011 | 1.006 |
| anhydromuropeptides recycling | -1.196 | 1.006 |
| NAD phosphorylation and dephosphorylation | 1.020 | 1.005 |
| glycine biosynthesis I | -1.211 | 1.005 |
| 2-oxoglutarate decarboxylation to succinyl-CoA | 1.007 | 1.005 |
| thiamine diphosphate biosynthesis I (E. coli) | 1.006 | 1.005 |
| L-threonine biosynthesis | 1.006 | 1.004 |
| urea cycle | 1.004 | 1.004 |
| L-tryptophan biosynthesis | 1.004 | 1.004 |
| queuosine biosynthesis | -1.221 | 1.004 |
| L-phenylalanine degradation I (aerobic) | 1.011 | 1.003 |
| inosine-5'-phosphate biosynthesis I | 1.004 | 1.003 |
| L-serine biosynthesis | 1.004 | 1.003 |
| L-glutamine degradation I | 1.002 | 1.002 |
| 3-dehydroquinate biosynthesis I | 1.002 | 1.002 |
| chorismate biosynthesis from 3-dehydroquinate | 1.002 | 1.002 |
| UMP biosynthesis | 1.003 | 1.002 |
| thioredoxin pathway | 1.003 | 1.002 |
| fatty acid elongation -- saturated | 1.003 | 1.002 |
| acetate conversion to acetyl-CoA | -1.004 | 1.002 |

| | Control surface I (Rubber elastomer) | Artificial biofilm |
|---|---|---|
| biotin-carboxyl carrier protein assembly | 1.003 | 1.001 |
| superoxide radicals degradation | 1.002 | 1.001 |
| pyrimidine deoxyribonucleotide phosphorylation | 1.002 | 1.001 |
| L-homoserine biosynthesis | 1.003 | 1.001 |
| guanosine ribonucleotides de novo biosynthesis | -1.202 | 1.001 |
| 5.6-dimethylbenzimidazole biosynthesis I (aerobic) | -1.243 | 1.001 |
| UDP-N-acetylmuramoyl-pentapeptide biosynthesis I (meso-diaminopimelate containing) | 1.002 | 1.001 |
| S-adenosyl-L-methionine biosynthesis | 1.003 | 1.000 |
| adenosine ribonucleotides de novo biosynthesis | 1.000 | 1.000 |
| tRNA charging | 1.000 | 1.000 |
| ethylmalonyl-CoA pathway | 25.376 | 0.000 |
| UTP and CTP dephosphorylation II | 11.842 | 0.000 |
| 4-chlorobenzoate degradation | 5.075 | 0.000 |
| (S)-acetoin biosynthesis | 2.497 | 0.000 |
| N-acetylglutaminylglutamine amide biosynthesis | 2.266 | 0.000 |
| D-allose degradation | 2.070 | 0.000 |
| TCA cycle III (animals) | 1.453 | 0.000 |
| pentachlorophenol degradation | 1.420 | 0.000 |
| (1.3)-beta-D-xylan degradation | 0.000 | 0.000 |
| (R)-acetoin biosynthesis I | 0.000 | 0.000 |
| (R)-cysteate degradation | 0.000 | 0.000 |
| (R.R)-butanediol biosynthesis | 0.000 | 0.000 |
| (R.R)-butanediol degradation | 0.000 | 0.000 |
| (S.S)-butanediol biosynthesis | 0.000 | 0.000 |
| (S.S)-butanediol degradation | 0.000 | 0.000 |
| 1.3-beta-D-glucan biosynthesis | 0.000 | 0.000 |
| 1.4-dihydroxy-2-naphthoate biosynthesis I | 0.000 | 0.000 |
| 1.4-dihydroxy-6-naphthoate biosynthesis I | 0.000 | 0.000 |
| 1.4-dihydroxy-6-naphthoate biosynthesis II | 0.000 | 0.000 |
| 2.2'-dihydroxybiphenyl degradation | 0.000 | 0.000 |
| 2.4-dichlorophenoxyacetate degradation | 0.000 | 0.000 |
| 2-chlorobenzoate degradation | 0.000 | 0.000 |
| 2-heptyl-3-hydroxy-4(1H)-quinolone biosynthesis | 0.000 | 0.000 |
| 2-keto-L-gulonate biosynthesis | 0.000 | 0.000 |
| 2-methylpropene degradation | 0.000 | 0.000 |
| 2-oxopentenoate degradation | 0.000 | 0.000 |
| 2-propylphenol degradation | 0.000 | 0.000 |
| 3.6-anhydro-alpha-L-galactopyranose degradation | 0.000 | 0.000 |
| 3-chlorobenzoate degradation II (via protocatechuate) | 0.000 | 0.000 |
| 3-methylarginine biosynthesis | 0.000 | 0.000 |
| 3-methylthiopropanoate biosynthesis | 0.000 | 0.000 |
| 3-oxoadipate degradation | 0.000 | 0.000 |
| 4.4'-diapolycopenedioate biosynthesis | 0.000 | 0.000 |
| 4-aminobutyrate degradation III | 0.000 | 0.000 |
| 4-hydroxymandelate degradation | 0.000 | 0.000 |
| 4-hydroxyphenylacetate degradation | 0.000 | 0.000 |
| 5.5'-dehydrodivanillate degradation | 0.000 | 0.000 |
| 5.6-dimethylbenzimidazole biosynthesis | 0.000 | 0.000 |
| 5.6-dimethylbenzimidazole biosynthesis II (anaerobic) | 0.000 | 0.000 |
| 8-amino-7-oxononanoate biosynthesis III | 0.000 | 0.000 |

| | Control surface I (Rubber elastomer) | Artificial biofilm |
|---|---|---|
| Bifidobacterium shunt | 0.000 | 0.000 |
| CMP-KDO biosynthesis II (from D-arabinose 5-phosphate) | 0.000 | 0.000 |
| CMP-legionaminate biosynthesis I | 0.000 | 0.000 |
| CMP-legionaminate biosynthesis II | 0.000 | 0.000 |
| D-arabinose degradation II | 0.000 | 0.000 |
| D-galacturonate degradation II | 0.000 | 0.000 |
| D-glucosaminate degradation | 0.000 | 0.000 |
| D-glucuronate degradation II | 0.000 | 0.000 |
| D-malate degradation | 0.000 | 0.000 |
| GDP-6-deoxy-D-talose biosynthesis | 0.000 | 0.000 |
| GDP-glucose biosynthesis II | 0.000 | 0.000 |
| L-1.2-propanediol degradation | 0.000 | 0.000 |
| L-alanine degradation I | 0.000 | 0.000 |
| L-arabinose degradation III | 0.000 | 0.000 |
| L-ascorbate biosynthesis III | 0.000 | 0.000 |
| L-carnitine degradation I | 0.000 | 0.000 |
| L-citrulline biosynthesis | 0.000 | 0.000 |
| L-cysteine biosynthesis III (from L-homocysteine) | 0.000 | 0.000 |
| L-galactonate degradation | 0.000 | 0.000 |
| L-glutamine biosynthesis II (tRNA-dependent) | 0.000 | 0.000 |
| L-glutamine biosynthesis III | 0.000 | 0.000 |
| L-histidine degradation III | 0.000 | 0.000 |
| L-lactaldehyde degradation (aerobic) | 0.000 | 0.000 |
| L-lysine degradation IV | 0.000 | 0.000 |
| L-lysine degradation V | 0.000 | 0.000 |
| L-lyxonate degradation | 0.000 | 0.000 |
| L-rhamnose degradation II | 0.000 | 0.000 |
| L-sorbose degradation | 0.000 | 0.000 |
| L-threonine degradation III (to methylglyoxal) | 0.000 | 0.000 |
| L-tryptophan degradation II (via pyruvate) | 0.000 | 0.000 |
| L-tryptophan degradation VI (via tryptamine) | 0.000 | 0.000 |
| L-tyrosine biosynthesis II | 0.000 | 0.000 |
| NAD biosynthesis III | 0.000 | 0.000 |
| NAD phosphorylation and dephosphorylation II (mammalian) | 0.000 | 0.000 |
| NADH to cytochrome bd oxidase electron transport II | 0.000 | 0.000 |
| NADH to cytochrome bo oxidase electron transfer II | 0.000 | 0.000 |
| NADH to dimethyl sulfoxide electron transfer | 0.000 | 0.000 |
| NADH to fumarate electron transfer | 0.000 | 0.000 |
| NADH to trimethylamine N-oxide electron transfer | 0.000 | 0.000 |
| S-methyl-5'-thioadenosine degradation III | 0.000 | 0.000 |
| TCA cycle IV (2-oxoglutarate decarboxylase) | 0.000 | 0.000 |
| TCA cycle VII (acetate-producers) | 0.000 | 0.000 |
| UDP-2.3-diacetamido-2.3-dideoxy-alpha-D-mannuronate biosynthesis | 0.000 | 0.000 |
| UDP-N.N'-diacetylbacillosamine biosynthesis | 0.000 | 0.000 |
| UDP-N-acetyl-D-galactosamine biosynthesis I | 0.000 | 0.000 |
| UDP-N-acetyl-D-glucosamine biosynthesis II | 0.000 | 0.000 |
| UDP-N-acetyl-alpha-D-galactosaminuronate biosynthesis | 0.000 | 0.000 |
| UDP-N-acetyl-beta-L-fucosamine biosynthesis | 0.000 | 0.000 |
| UDP-N-acetylmuramoyl-pentapeptide biosynthesis I (meso-DAP-containing) | 0.000 | 0.000 |
| UDP-N-acetylmuramoyl-pentapeptide biosynthesis II (lysine-containing) | 0.000 | 0.000 |
| acetate formation from acetyl-CoA II | 0.000 | 0.000 |

| | Control surface I (Rubber elastomer) | Artificial biofilm |
|---|---|---|
| acetoacetate degradation (to acetyl CoA) | 0.000 | 0.000 |
| acrylonitrile degradation I | 0.000 | 0.000 |
| acrylonitrile degradation II | 0.000 | 0.000 |
| acyl carrier protein metabolism II (mammalian) | 0.000 | 0.000 |
| adenine and adenosine salvage IV | 0.000 | 0.000 |
| aerobactin biosynthesis | 0.000 | 0.000 |
| aerobic respiration II (cytochrome c) (yeast) | 0.000 | 0.000 |
| alanine biosynthesis I | 0.000 | 0.000 |
| alanine biosynthesis III | 0.000 | 0.000 |
| aldoxime degradation | 0.000 | 0.000 |
| alginate biosynthesis II (bacterial) | 0.000 | 0.000 |
| all-trans-decaprenyl diphosphate biosynthesis | 0.000 | 0.000 |
| alpha-diglucosyldiacylglycerol biosynthesis | 0.000 | 0.000 |
| aminopropanol phosphate biosynthesis I | 0.000 | 0.000 |
| ammonia oxidation I (aerobic) | 0.000 | 0.000 |
| arachidonate biosynthesis II (bacteria) | 0.000 | 0.000 |
| arginine biosynthesis II (acetyl cycle) | 0.000 | 0.000 |
| arginine degradation I (arginase pathway) | 0.000 | 0.000 |
| arginine degradation VI (arginase 2 pathway) | 0.000 | 0.000 |
| arginine degradation VII (arginase 3 pathway) | 0.000 | 0.000 |
| arsenate detoxification III (mycothiol) | 0.000 | 0.000 |
| asparagine biosynthesis III (tRNA-dependent) | 0.000 | 0.000 |
| aspartate biosynthesis | 0.000 | 0.000 |
| aspartate degradation I | 0.000 | 0.000 |
| aspartate degradation II | 0.000 | 0.000 |
| bacillithiol biosynthesis | 0.000 | 0.000 |
| base-degraded thiamin salvage | 0.000 | 0.000 |
| benzoyl-CoA degradation II (anaerobic) | 0.000 | 0.000 |
| beta-alanine degradation I | 0.000 | 0.000 |
| beta-alanine degradation II | 0.000 | 0.000 |
| beta-carotene biosynthesis | 0.000 | 0.000 |
| bile acids degradation | 0.000 | 0.000 |
| carbazole degradation | 0.000 | 0.000 |
| carbon monoxide oxidation to CO2 | 0.000 | 0.000 |
| catechol degradation to 2-oxopent-4-enoate II | 0.000 | 0.000 |
| cephamycin C biosynthesis | 0.000 | 0.000 |
| chitin degradation to ethanol | 0.000 | 0.000 |
| chlorogenic acid degradation | 0.000 | 0.000 |
| chlorophyllide a biosynthesis I (aerobic. light-dependent) | 0.000 | 0.000 |
| cholate degradation (bacteria. anaerobic) | 0.000 | 0.000 |
| chondroitin sulfate degradation I (bacterial) | 0.000 | 0.000 |
| cis-dodecenoyl biosynthesis | 0.000 | 0.000 |
| citronellol degradation | 0.000 | 0.000 |
| citrulline biosynthesis | 0.000 | 0.000 |
| citrulline-nitric oxide cycle | 0.000 | 0.000 |
| cob(II)yrinate a.c-diamide biosynthesis I (early cobalt insertion) | 0.000 | 0.000 |
| coenzyme A biosynthesis II (mammalian) | 0.000 | 0.000 |
| curcumin degradation | 0.000 | 0.000 |
| dTDP-D-olivose. dTDP-D-oliose and dTDP-D-mycarose biosynthesis | 0.000 | 0.000 |
| dTDP-N-acetylthomosamine biosynthesis | 0.000 | 0.000 |
| daidzin and daidzein degradation | 0.000 | 0.000 |

| | Control surface I (Rubber elastomer) | Artificial biofilm |
|---|---|---|
| deacetylcephalosporin C biosynthesis | 0.000 | 0.000 |
| dermatan sulfate degradation I (bacterial) | 0.000 | 0.000 |
| dibenzothiophene desulfurization | 0.000 | 0.000 |
| dimethylsulfoniopropanoate degradation I (cleavage) | 0.000 | 0.000 |
| erythromycin A biosynthesis | 0.000 | 0.000 |
| fatty acid activation | 0.000 | 0.000 |
| fatty acid beta-oxidation II (peroxisome) | 0.000 | 0.000 |
| fatty acid biosynthesis initiation III | 0.000 | 0.000 |
| fatty acid salvage | 0.000 | 0.000 |
| ferulate degradation | 0.000 | 0.000 |
| flaviolin dimer and mompain biosynthesis | 0.000 | 0.000 |
| fluoroacetate and fluorothreonine biosynthesis | 0.000 | 0.000 |
| formaldehyde oxidation V (H4MPT pathway) | 0.000 | 0.000 |
| formate reduction to 5.10-methylenetetrahydrofolate | 0.000 | 0.000 |
| formate to dimethyl sulfoxide electron transfer | 0.000 | 0.000 |
| formate to trimethylamine N-oxide electron transfer | 0.000 | 0.000 |
| galactitol degradation | 0.000 | 0.000 |
| gallate degradation I | 0.000 | 0.000 |
| gamma-resorcylate degradation I | 0.000 | 0.000 |
| gentisate degradation I | 0.000 | 0.000 |
| glucose degradation (oxidative) | 0.000 | 0.000 |
| glucosylglycerate biosynthesis I | 0.000 | 0.000 |
| glucosylglycerate biosynthesis II | 0.000 | 0.000 |
| glucuronoarabinoxylan degradation | 0.000 | 0.000 |
| glutamate biosynthesis I | 0.000 | 0.000 |
| glutamate biosynthesis II | 0.000 | 0.000 |
| glutamate biosynthesis III | 0.000 | 0.000 |
| glutamate degradation I | 0.000 | 0.000 |
| glutamate degradation II | 0.000 | 0.000 |
| glutamate degradation X | 0.000 | 0.000 |
| glutamine degradation I | 0.000 | 0.000 |
| glutamine degradation II | 0.000 | 0.000 |
| glutathione redox reactions II | 0.000 | 0.000 |
| glycerol degradation II | 0.000 | 0.000 |
| glycine betaine biosynthesis IV (from glycine) | 0.000 | 0.000 |
| glycine betaine degradation II (mammalian) | 0.000 | 0.000 |
| glycine biosynthesis IV | 0.000 | 0.000 |
| glycocholate metabolism (bacteria) | 0.000 | 0.000 |
| glycogen degradation I | 0.000 | 0.000 |
| glycogenolysis II | 0.000 | 0.000 |
| glycolate and glyoxylate degradation II | 0.000 | 0.000 |
| glycolysis IV (plant cytosol) | 0.000 | 0.000 |
| heparan sulfate degradation | 0.000 | 0.000 |
| hexaprenyl diphosphate biosynthesis | 0.000 | 0.000 |
| histidine biosynthesis | 0.000 | 0.000 |
| histidine degradation II | 0.000 | 0.000 |
| homocysteine biosynthesis | 0.000 | 0.000 |
| homoserine biosynthesis | 0.000 | 0.000 |
| homospermidine biosynthesis | 0.000 | 0.000 |
| hopanoid biosynthesis (bacteria) | 0.000 | 0.000 |
| hydrogen oxidation II (aerobic. NAD) | 0.000 | 0.000 |

| | **Control surface I (Rubber elastomer)** | **Artificial biofilm** |
|---|---|---|
| hydrogen oxidation III (anaerobic. NADP) | 0.000 | 0.000 |
| hydrogen production II | 0.000 | 0.000 |
| hydrogen production III | 0.000 | 0.000 |
| hydrogen production VI | 0.000 | 0.000 |
| hydrogen sulfide biosynthesis I | 0.000 | 0.000 |
| hydrogen to dimethyl sulfoxide electron transfer | 0.000 | 0.000 |
| hydrogen to trimethylamine N-oxide electron transfer | 0.000 | 0.000 |
| incomplete reductive TCA cycle | 0.000 | 0.000 |
| iota-carrageenan degradation | 0.000 | 0.000 |
| isoleucine biosynthesis I (from threonine) | 0.000 | 0.000 |
| isopenicillin N biosynthesis | 0.000 | 0.000 |
| itaconate biosynthesis | 0.000 | 0.000 |
| itaconate degradation | 0.000 | 0.000 |
| kanosamine biosynthesis II | 0.000 | 0.000 |
| kappa-carrageenan degradation | 0.000 | 0.000 |
| ketogenesis | 0.000 | 0.000 |
| ketolysis | 0.000 | 0.000 |
| kojibiose degradation | 0.000 | 0.000 |
| lactose and galactose degradation I | 0.000 | 0.000 |
| leucine biosynthesis | 0.000 | 0.000 |
| leucine degradation I | 0.000 | 0.000 |
| leucine degradation IV | 0.000 | 0.000 |
| limonene degradation III (to perillate) | 0.000 | 0.000 |
| lincomycin biosynthesis | 0.000 | 0.000 |
| linezolid resistance | 0.000 | 0.000 |
| lipoate biosynthesis and incorporation (glycine cleavage system. yeast) | 0.000 | 0.000 |
| lipoate biosynthesis and incorporation III (Bacillus) | 0.000 | 0.000 |
| lipoate salvage II | 0.000 | 0.000 |
| lysine biosynthesis I | 0.000 | 0.000 |
| lysine biosynthesis III | 0.000 | 0.000 |
| lysine degradation I | 0.000 | 0.000 |
| lysine fermentation to acetate and butyrate | 0.000 | 0.000 |
| malonate decarboxylase activation | 0.000 | 0.000 |
| manganese oxidation II | 0.000 | 0.000 |
| mannan degradation | 0.000 | 0.000 |
| mannosylglucosylglycerate biosynthesis I | 0.000 | 0.000 |
| mannosylglucosylglycerate biosynthesis II | 0.000 | 0.000 |
| mannosylglycerate biosynthesis II | 0.000 | 0.000 |
| methanesulfonate degradation | 0.000 | 0.000 |
| methanol oxidation to formaldehyde III | 0.000 | 0.000 |
| methanol oxidation to formaldehyde IV | 0.000 | 0.000 |
| methionine biosynthesis I | 0.000 | 0.000 |
| methionine degradation I (to homocysteine) | 0.000 | 0.000 |
| methylamine degradation II | 0.000 | 0.000 |
| methylerythritol phosphate pathway I | 0.000 | 0.000 |
| methylglyoxal degradation II | 0.000 | 0.000 |
| methylphosphonate degradation II | 0.000 | 0.000 |
| methylthiopropanoate degradation I (cleavage) | 0.000 | 0.000 |
| mycocyclosin biosynthesis | 0.000 | 0.000 |
| neurosporene biosynthesis | 0.000 | 0.000 |
| nicotinate degradation I | 0.000 | 0.000 |

| | Control surface I (Rubber elastomer) | Artificial biofilm |
|---|---|---|
| nicotine degradation II (pyrrolidine pathway) | 0.000 | 0.000 |
| nitrate reduction III (dissimilatory) | 0.000 | 0.000 |
| nitrate reduction IV (dissimilatory) | 0.000 | 0.000 |
| nitrate reduction V (assimilatory) | 0.000 | 0.000 |
| nitrate reduction VI (assimilatory) | 0.000 | 0.000 |
| nitrite-dependent anaerobic methane oxidation | 0.000 | 0.000 |
| nitrobenzene degradation I | 0.000 | 0.000 |
| nitrogen fixation II (flavodoxin) | 0.000 | 0.000 |
| nonaprenyl diphosphate biosynthesis I | 0.000 | 0.000 |
| o-diquinones biosynthesis | 0.000 | 0.000 |
| oleandomycin activation/inactivation | 0.000 | 0.000 |
| oligomeric urushiol biosynthesis | 0.000 | 0.000 |
| ornithine biosynthesis | 0.000 | 0.000 |
| ornithine de novo biosynthesis | 0.000 | 0.000 |
| ornithine degradation I (proline biosynthesis) | 0.000 | 0.000 |
| oxalate degradation I | 0.000 | 0.000 |
| oxalate degradation IV | 0.000 | 0.000 |
| oxalate degradation V | 0.000 | 0.000 |
| palmitoleate biosynthesis I | 0.000 | 0.000 |
| palmitoleate biosynthesis II (plants and bacteria) | 0.000 | 0.000 |
| partial TCA cycle (obligate autotrophs) | 0.000 | 0.000 |
| pectin degradation I | 0.000 | 0.000 |
| perchlorate reduction | 0.000 | 0.000 |
| phenol degradation I (aerobic) | 0.000 | 0.000 |
| phenylacetate degradation II (anaerobic) | 0.000 | 0.000 |
| phenylalanine biosynthesis I | 0.000 | 0.000 |
| phenylalanine degradation I (aerobic) | 0.000 | 0.000 |
| phenylalanine degradation V | 0.000 | 0.000 |
| phenylethylamine degradation I | 0.000 | 0.000 |
| phosphatidylcholine biosynthesis I | 0.000 | 0.000 |
| phosphatidylcholine biosynthesis VII | 0.000 | 0.000 |
| phosphatidylcholine resynthesis via glycerophosphocholine | 0.000 | 0.000 |
| phytol degradation | 0.000 | 0.000 |
| polyhydroxybutyrate biosynthesis | 0.000 | 0.000 |
| polyvinyl alcohol degradation | 0.000 | 0.000 |
| proline biosynthesis I | 0.000 | 0.000 |
| proline degradation | 0.000 | 0.000 |
| propionyl CoA degradation | 0.000 | 0.000 |
| protein N-glycosylation (bacterial) | 0.000 | 0.000 |
| protocatechuate degradation I (meta-cleavage pathway) | 0.000 | 0.000 |
| pulcherrimin biosynthesis | 0.000 | 0.000 |
| pyocyanin biosynthesis | 0.000 | 0.000 |
| pyridoxal 5'-phosphate biosynthesis II | 0.000 | 0.000 |
| pyrimidine deoxyribonucleotides biosynthesis from CTP | 0.000 | 0.000 |
| pyrimidine ribonucleosides salvage II | 0.000 | 0.000 |
| pyrimidine ribonucleosides salvage III | 0.000 | 0.000 |
| pyrrolnitrin biosynthesis | 0.000 | 0.000 |
| pyruvate fermentation to acetone | 0.000 | 0.000 |
| pyruvate fermentation to ethanol II | 0.000 | 0.000 |
| pyruvate fermentation to ethanol III | 0.000 | 0.000 |
| pyruvate fermentation to lactate | 0.000 | 0.000 |

| | Control surface I (Rubber elastomer) | Artificial biofilm |
|---|---|---|
| pyruvate fermentation to propionate I | 0.000 | 0.000 |
| pyruvate to cytochrome bd terminal oxidase electron transfer | 0.000 | 0.000 |
| pyruvate to cytochrome bo oxidase electron transfer | 0.000 | 0.000 |
| rhamnogalacturonan type I degradation II (bacteria) | 0.000 | 0.000 |
| rhamnolipid biosynthesis | 0.000 | 0.000 |
| seed germination protein turnover | 0.000 | 0.000 |
| selenocysteine biosynthesis I (bacteria) | 0.000 | 0.000 |
| serine biosynthesis | 0.000 | 0.000 |
| shikimate degradation I | 0.000 | 0.000 |
| spermidine biosynthesis II | 0.000 | 0.000 |
| spermine and spermidine degradation II | 0.000 | 0.000 |
| spermine biosynthesis | 0.000 | 0.000 |
| sphingomyelin metabolism | 0.000 | 0.000 |
| starch degradation III | 0.000 | 0.000 |
| stearate biosynthesis III (fungi) | 0.000 | 0.000 |
| sucrose biosynthesis II | 0.000 | 0.000 |
| sucrose degradation II (sucrose synthase) | 0.000 | 0.000 |
| sulfate reduction II (assimilatory) | 0.000 | 0.000 |
| sulfate reduction IV (dissimilatory) | 0.000 | 0.000 |
| sulfate reduction V (dissimilatory) | 0.000 | 0.000 |
| sulfite oxidation I (sulfite oxidoreductase) | 0.000 | 0.000 |
| sulfite oxidation III | 0.000 | 0.000 |
| sulfoacetate degradation | 0.000 | 0.000 |
| sulfoquinovose degradation I | 0.000 | 0.000 |
| taurine biosynthesis | 0.000 | 0.000 |
| tetrachloroethene degradation | 0.000 | 0.000 |
| tetrathionate oxidation | 0.000 | 0.000 |
| thiamin diphosphate biosynthesis I (E. coli) | 0.000 | 0.000 |
| thiamin diphosphate biosynthesis II (Bacillus) | 0.000 | 0.000 |
| thiamin salvage IV (yeast) | 0.000 | 0.000 |
| thiamine diphosphate biosynthesis III (Staphylococcus) | 0.000 | 0.000 |
| thiamine triphosphate metabolism | 0.000 | 0.000 |
| thiazole biosynthesis I (E. coli) | 0.000 | 0.000 |
| thiazole biosynthesis II (Bacillus) | 0.000 | 0.000 |
| thiocyanate degradation II | 0.000 | 0.000 |
| thiosulfate oxidation I (to tetrathionate) | 0.000 | 0.000 |
| threonine biosynthesis from homoserine | 0.000 | 0.000 |
| trans-4-hydroxy-L-proline degradation II | 0.000 | 0.000 |
| trehalose degradation IV | 0.000 | 0.000 |
| triclosan resistance | 0.000 | 0.000 |
| tryptophan biosynthesis | 0.000 | 0.000 |
| tryptophan degradation I (via anthranilate) | 0.000 | 0.000 |
| tryptophan degradation to 2-amino-3-carboxymuconate semialdehyde | 0.000 | 0.000 |
| tyrosine biosynthesis I | 0.000 | 0.000 |
| tyrosine biosynthesis IV | 0.000 | 0.000 |
| unsaturated. even numbered fatty acid beta-oxidation | 0.000 | 0.000 |
| uracil degradation II (oxidative) | 0.000 | 0.000 |
| uracil degradation III | 0.000 | 0.000 |
| urate degradation to allantoin II | 0.000 | 0.000 |
| valine biosynthesis | 0.000 | 0.000 |
| vancomycin resistance I | 0.000 | 0.000 |

| | Control surface I (Rubber elastomer) | Artificial biofilm |
|---|---|---|
| vancomycin resistance II | 0.000 | 0.000 |
| very long chain fatty acid biosynthesis I | 0.000 | 0.000 |
| wax esters biosynthesis II | 0.000 | 0.000 |
| wound-induced proteolysis I | 0.000 | 0.000 |
| xanthan biosynthesis | 0.000 | 0.000 |
| xylose degradation III | 0.000 | 0.000 |
| zeaxanthin-beta-D-diglucoside biosynthesis | 0.000 | 0.000 |
| dimethyl sulfide degradation III (oxidation) | -1.792 | 0.000 |
| sulfoquinovosyl diacylglycerol biosynthesis | -2.034 | 0.000 |
| guanosine nucleotides degradation II | -2.696 | 0.000 |
| cis-genanyl-CoA degradation | -3.604 | 0.000 |
| L-lysine biosynthesis VI | -5.311 | 0.000 |
| choline biosynthesis III | -9.927 | 0.000 |
| acetone degradation II (to acetoacetate) | -11.687 | 0.000 |
| L-arginine biosynthesis III (via N-acetyl-L-citrulline) | -15.975 | 0.000 |
| salicylate biosynthesis I | -16.027 | 0.000 |
| L-ascorbate degradation II (bacterial. aerobic) | -20.409 | 0.000 |
| UDP-D-galacturonate biosynthesis I (from UDP-D-glucuronate) | -29.388 | 0.000 |
| pectin degradation III | -32.440 | 0.000 |
| paraoxon degradation | -38.775 | 0.000 |
| parathion degradation | -38.775 | 0.000 |
| acyl-ACP thioesterase pathway | -52.471 | 0.000 |
| photosynthesis light reactions | -54.614 | 0.000 |
| PRPP biosynthesis I | -1.011 | -1.000 |
| CMP phosphorylation | 1.001 | -1.000 |
| UTP and CTP de novo biosynthesis | 1.001 | -1.000 |
| urea degradation II | 1.074 | -1.002 |
| formaldehyde oxidation II (glutathione-dependent) | 1.291 | -1.003 |
| N10-formyl-tetrahydrofolate biosynthesis | 1.168 | -1.026 |
| D-sorbitol degradation I | 1.663 | -1.028 |
| dimethyl sulfide degradation II (oxidation) | 0.000 | -1.037 |
| gluconeogenesis I | 1.142 | -1.040 |
| oleate beta-oxidation | 1.500 | -1.051 |
| L-arginine biosynthesis II (acetyl cycle) | 1.230 | -1.070 |
| 2-oxoisovalerate decarboxylation to isobutanoyl-CoA | -1.001 | -1.078 |
| heme biosynthesis II (anaerobic) | -2.347 | -1.088 |
| adenine and adenosine salvage I | -1.028 | -1.109 |
| putrescine biosynthesis III | -1.886 | -1.114 |
| cyclopropane fatty acid (CFA) biosynthesis | -1.028 | -1.115 |
| uracil degradation I (reductive) | 1.646 | -1.125 |
| muropeptide degradation | 0.000 | -1.127 |
| pyrimidine ribonucleosides degradation | -4.689 | -1.133 |
| GDP-mannose biosynthesis | -1.095 | -1.138 |
| formaldehyde assimilation I (serine pathway) | 1.251 | -1.143 |
| NAD phosphorylation and transhydrogenation | 1.214 | -1.150 |
| beta-D-glucuronide and D-glucuronate degradation | 2.380 | -1.170 |
| di-trans.poly-cis-undecaprenyl phosphate biosynthesis | 1.130 | -1.187 |
| D-galacturonate degradation I | 1.384 | -1.197 |
| tetrahydrofolate biosynthesis | 1.086 | -1.204 |
| glycine cleavage | 1.035 | -1.209 |
| L-isoleucine degradation I | 1.343 | -1.210 |

| | Control surface I (Rubber elastomer) | Artificial biofilm |
|---|---|---|
| L-lysine fermentation to acetate and butanoate | 0.000 | -1.214 |
| L-aspartate biosynthesis | 1.080 | -1.217 |
| L-aspartate degradation I | 1.080 | -1.217 |
| bis(guanylyl molybdenum cofactor) biosynthesis | -1.521 | -1.218 |
| sulfate activation for sulfonation | 1.229 | -1.244 |
| UDP-D-galactose biosynthesis | 1.021 | -1.247 |
| pyrimidine deoxyribonucleotides dephosphorylation | -1.148 | -1.252 |
| L-asparagine biosynthesis III (tRNA-dependent) | 1.649 | -1.255 |
| folate polyglutamylation | 1.039 | -1.258 |
| malonate degradation I (biotin-independent) | -2.613 | -1.261 |
| adenosylcobalamin biosynthesis from cobyrinate a.c-diamide II | 1.602 | -1.264 |
| menaquinol-7 biosynthesis | -1.588 | -1.265 |
| menaquinol-9 biosynthesis | -1.583 | -1.272 |
| menaquinol-6 biosynthesis | -1.586 | -1.274 |
| taurine degradation IV | -97.274 | -1.291 |
| trehalose biosynthesis V | 1.196 | -1.294 |
| arsenate detoxification II (glutaredoxin) | -3.215 | -1.311 |
| 5-aminoimidazole ribonucleotide biosynthesis I | -1.169 | -1.318 |
| L-glutamate biosynthesis II | -1.109 | -1.322 |
| L-glutamate degradation X | -1.109 | -1.322 |
| lipoate biosynthesis and incorporation I | -1.207 | -1.324 |
| NAD biosynthesis from 2-amino-3-carboxymuconate semialdehyde | -5.077 | -1.325 |
| oxidized GTP and dGTP detoxification | -4.372 | -1.357 |
| glucose and glucose-1-phosphate degradation | -3.096 | -1.369 |
| NADH repair | -2.610 | -1.394 |
| cyanate degradation | -1.010 | -1.457 |
| folate transformations I | -1.001 | -1.457 |
| TCA cycle VIII (helicobacter) | -4.107 | -1.477 |
| catechol degradation to beta-ketoadipate | -19.782 | -1.506 |
| menaquinol-8 biosynthesis | 1.259 | -1.573 |
| formaldehyde assimilation II (RuMP Cycle) | -105.073 | -1.597 |
| siroheme biosynthesis | -3.936 | -1.615 |
| CDP-diacylglycerol biosynthesis I | -2.383 | -1.658 |
| urea degradation I | -3.357 | -1.674 |
| L-asparagine biosynthesis II | -2.156 | -1.685 |
| L-asparagine biosynthesis I | -2.155 | -1.686 |
| oxalate degradation II | -6.078 | -1.713 |
| adenine salvage | 1.459 | -1.755 |
| guanine and guanosine salvage III | 0.000 | -1.818 |
| xylose degradation I | 1.568 | -1.826 |
| vanillin and vanillate degradation I | -3.698 | -1.872 |
| cob(II)yrinate a.c-diamide biosynthesis II (late cobalt incorporation) | 1.178 | -1.922 |
| cinnamate and 3-hydroxycinnamate degradation to 2-oxopent-4-enoate | -5.674 | -1.924 |
| D-mannose degradation | 1.068 | -1.935 |
| coenzyme A biosynthesis I | 1.530 | -1.958 |
| adenine and adenosine salvage V | -84.179 | -2.103 |
| pyridoxal 5'-phosphate salvage I | 13.342 | -2.196 |
| glycogen degradation II | -1.890 | -2.226 |
| L-threonine degradation II | -3.874 | -2.246 |
| L-methionine biosynthesis I | 1.419 | -2.256 |
| L-methionine degradation I (to L-homocysteine) | -1.401 | -2.373 |

| | Control surface I (Rubber elastomer) | Artificial biofilm |
|---|---|---|
| L-idonate degradation | 4.503 | -2.555 |
| D-galactonate degradation | 1.496 | -2.570 |
| adenosylcobalamin salvage from cobinamide I | 39.999 | -2.602 |
| sulfur reduction II (via polysulfide) | -117.403 | -2.627 |
| autoinducer AI-2 degradation | -3.786 | -2.636 |
| formate assimilation into 5.10-methylenetetrahydrofolate | 1.392 | -2.705 |
| D-galactarate degradation II | 3.876 | -2.915 |
| S-adenosyl-L-methionine cycle II | 1.208 | -2.923 |
| pyrimidine deoxyribonucleosides salvage | -1.060 | -3.329 |
| nylon-6 oligomer degradation | -2.059 | -3.334 |
| 3-chlorobenzoate degradation III (via gentisate) | 93.658 | -3.370 |
| salicylate degradation IV | -2.400 | -3.464 |
| L-threitol degradation | 0.000 | -3.706 |
| erythritol degradation II | 0.000 | -3.706 |
| L-histidine degradation II | 1.758 | -3.722 |
| pyruvate decarboxylation to acetyl CoA | -1.032 | -4.045 |
| L-alanine biosynthesis I | -1.114 | -4.723 |
| fructoselysine and psicoselysine degradation | -14.465 | -4.836 |
| glycine biosynthesis III | -1.073 | -4.876 |
| CMP-3-deoxy-D-manno-octulosonate biosynthesis | -192.127 | -4.979 |
| hydrogen to fumarate electron transfer | 2.436 | -5.501 |
| histamine biosynthesis | 0.000 | -5.909 |
| beta-alanine biosynthesis II | 1.877 | -5.971 |
| aromatic biogenic amine degradation (bacteria) | 51.131 | -6.173 |
| pyrroloquinoline quinone biosynthesis | -1.497 | -7.158 |
| sulfolactate degradation III | -1.263 | -7.855 |
| triacylglycerol degradation | 0.000 | -7.910 |
| L-dopachrome biosynthesis | 38.213 | -8.268 |
| L-lysine biosynthesis III | 0.000 | -8.523 |
| quinate degradation I | 0.000 | -8.770 |
| 4-hydroxyacetophenone degradation | 1.542 | -8.810 |
| testosterone and androsterone degradation to androstendione | 0.000 | -9.018 |
| heptaprenyl diphosphate biosynthesis | 0.000 | -9.615 |
| agarose degradation | 0.000 | -11.710 |
| porphyran degradation | 0.000 | -11.710 |
| spirilloxanthin and 2.2'-diketo-spirilloxanthin biosynthesis | 1.646 | -11.866 |
| phosphatidylcholine biosynthesis V | -12.595 | -11.890 |
| trehalose degradation II (trehalase) | -6.355 | -11.950 |
| trehalose degradation VI (periplasmic) | -6.355 | -11.950 |
| L-rhamnose degradation I | 1.186 | -11.968 |
| sulfoacetaldehyde degradation II | 27.377 | -12.750 |
| pyruvate fermentation to ethanol I | 0.000 | -13.129 |
| phospholipases | 0.000 | -13.383 |
| D-galactose degradation V (Leloir pathway) | 0.000 | -13.433 |
| CMP-N-acetylneuraminate biosynthesis II (bacteria) | 2.585 | -14.224 |
| hyaluronan degradation | 0.000 | -14.308 |
| putrescine biosynthesis IV | 0.000 | -15.377 |
| PRPP biosynthesis II | -3.731 | -15.776 |
| formaldehyde oxidation I | 0.000 | -16.902 |
| phosphate acquisition | -7.202 | -17.243 |
| C4 photosynthetic carbon assimilation cycle. NAD-ME type | 0.000 | -18.068 |

| | Control surface I (Rubber elastomer) | Artificial biofilm |
|---|---|---|
| glycerol-3-phosphate to fumarate electron transfer | -30.138 | -18.155 |
| fructan biosynthesis | -1.835 | -18.346 |
| arsenite oxidation II (respiratory) | 4.275 | -18.844 |
| GDP-D-perosamine biosynthesis | 0.000 | -19.145 |
| glycerol-3-phosphate to cytochrome bo oxidase electron transfer | -1.857 | -19.470 |
| L-lysine degradation I | 1.934 | -22.235 |
| phenylmercury acetate degradation | 3.333 | -23.187 |
| fatty acid biosynthesis initiation I | -4.311 | -24.207 |
| glutathione amide metabolism | 0.000 | -24.907 |
| pentose phosphate pathway (oxidative branch) | 1.781 | -25.322 |
| naphthalene degradation (aerobic) | -5.150 | -25.604 |
| spheroidene and spheroidenone biosynthesis | 1.498 | -26.201 |
| sulfur reduction I | 0.000 | -27.049 |
| glutaryl-CoA degradation | 0.000 | -27.493 |
| trehalose biosynthesis IV | 1.521 | -27.966 |
| sulfoacetaldehyde degradation I | 1.833 | -27.989 |
| sucrose degradation IV (sucrose phosphorylase) | 0.000 | -28.442 |
| hypotaurine degradation | 4.612 | -29.011 |
| taurine degradation I | 4.612 | -29.011 |
| adenine and adenosine salvage VI | 1.671 | -29.266 |
| diacylglyceryl-N.N.N-trimethylhomoserine biosynthesis | -3.453 | -29.593 |
| lactose degradation III | -1.016 | -30.317 |
| sucrose degradation III (sucrose invertase) | -1.148 | -32.049 |
| L-methionine degradation II | 1.590 | -32.912 |
| dimethylsulfoniopropanoate degradation III (demethylation) | 2.999 | -33.816 |
| acrylate degradation | 1.490 | -34.860 |
| L-ornithine biosynthesis II | -249.008 | -35.680 |
| NADH to cytochrome bo oxidase electron transfer I | -5.679 | -36.539 |
| L-proline biosynthesis III | -259.366 | -37.164 |
| 2.4-dichlorotoluene degradation | 4.853 | -39.794 |
| 2.5-dichlorotoluene degradation | 4.853 | -39.794 |
| 3.4-dichlorotoluene degradation | 4.853 | -39.794 |
| 4.5-dichlorocatechol degradation | 4.853 | -39.794 |
| 5-chloro-3-methyl-catechol degradation | 4.853 | -39.794 |
| autoinducer AI-1 biosynthesis | 1.865 | -39.854 |
| allantoin degradation to ureidoglycolate I (urea producing) | -60.706 | -40.215 |
| cellulose biosynthesis | -11.321 | -40.952 |
| octane oxidation | 1.372 | -41.011 |
| thiamine salvage III | 1.918 | -43.005 |
| pyrimidine deoxyribonucleotides de novo biosynthesis III | 1.671 | -43.409 |
| trehalose biosynthesis I | -6.465 | -44.861 |
| adenosine nucleotides degradation III | 1.093 | -45.202 |
| citrate degradation | -8.344 | -46.896 |
| ethene and chloroethene degradation | 1.909 | -47.004 |
| propene degradation | 1.909 | -47.004 |
| S-methyl-5'-thioadenosine degradation II | 1.331 | -47.067 |
| geranylgeranyl diphosphate biosynthesis | 1.127 | -47.552 |
| nitrate reduction X (periplasmic. dissimilatory) | -2.816 | -48.865 |
| (5Z)-dodec-5-enoate biosynthesis | -2.376 | -52.224 |
| oleate biosynthesis IV (anaerobic) | -2.376 | -52.224 |
| 3.8-divinyl-chlorophyllide a biosynthesis I (aerobic. light-dependent) | 1.038 | -54.307 |

| | Control surface I (Rubber elastomer) | Artificial biofilm |
|---|---|---|
| 3.8-divinyl-chlorophyllide a biosynthesis III (aerobic. light independent) | 1.038 | -54.307 |
| ethylene biosynthesis III (microbes) | 0.000 | -54.652 |
| erythromycin D biosynthesis | 0.000 | -55.087 |
| tetrapyrrole biosynthesis II (from glycine) | -2.023 | -57.182 |
| heparin degradation | 1.072 | -57.274 |
| glycine betaine degradation I | -2.022 | -58.943 |
| NAD salvage pathway I | 1.680 | -59.550 |
| L-glutamate degradation VI (to pyruvate) | -2.094 | -60.373 |
| pyruvate fermentation to propanoate I | 1.125 | -60.542 |
| glycogen biosynthesis I (from ADP-D-Glucose) | -1.401 | -61.879 |
| formate oxidation to CO2 | -1.540 | -61.903 |
| 4-hydroxybenzoate biosynthesis II (microbes) | 0.000 | -66.248 |
| 2-aminoethylphosphonate degradation II | -1.594 | -70.226 |
| propanoyl CoA degradation I | -2.200 | -70.837 |
| methylphosphonate degradation I | -2.716 | -76.945 |
| phosphonoacetate degradation | -1.673 | -77.203 |
| hydrogen oxidation I (aerobic) | -2.448 | -77.778 |
| acetoin degradation | -2.761 | -79.597 |
| L-tyrosine biosynthesis III | -1.183 | -82.504 |
| two-component alkanesulfonate monooxygenase | -1.273 | -85.703 |
| methylerythritol phosphate pathway II | 1.028 | -86.525 |
| adenosylcobalamin biosynthesis from cobyrinate a.c-diamide I | -2.633 | -86.836 |
| Calvin-Benson-Bassham cycle | -3.663 | -88.216 |
| urate degradation to allantoin I | -1.195 | -89.051 |
| maltose degradation | -395.985 | -90.415 |
| alginate degradation | -20.542 | -91.307 |
| sulfolactate degradation II | -2.587 | -91.761 |
| propanoyl-CoA degradation II | -1.218 | -92.426 |
| purine deoxyribonucleosides salvage | -216.875 | -96.680 |
| GDP-L-fucose biosynthesis I (from GDP-D-mannose) | -1.586 | -99.567 |
| nitrogen fixation I (ferredoxin) | -7.067 | -100.343 |
| pyruvate fermentation to acetate II | -3.625 | -101.801 |
| cyanophycin metabolism | -6.908 | -106.246 |
| glutathione-peroxide redox reactions | -3.759 | -113.106 |
| L-arginine degradation VI (arginase 2 pathway) | -338.302 | -113.108 |
| nitrate reduction IX (dissimilatory) | -2.970 | -114.532 |
| reductive acetyl coenzyme A pathway I (homoacetogenic bacteria) | -1.503 | -115.770 |
| cholesterol degradation to androstenedione II (cholesterol dehydrogenase) | -1.508 | -116.092 |
| fluoroacetate degradation | -1.508 | -116.139 |
| aerobic respiration I (cytochrome c) | -3.974 | -116.697 |
| androstenedione degradation | -1.551 | -116.999 |
| L-arginine degradation I (arginase pathway) | -245.039 | -117.037 |
| (R)-acetoin biosynthesis II | -295.773 | -117.273 |
| NADH to cytochrome bd oxidase electron transfer I | -4.702 | -118.091 |
| mevalonate pathway I | -247.463 | -118.195 |
| glycerol degradation V | 0.000 | -120.073 |
| limonene degradation I (D-limonene) | -37.785 | -125.097 |
| limonene degradation II (L-limonene) | -37.785 | -125.097 |
| adenine and adenosine salvage II | -475.238 | -127.112 |
| pseudouridine degradation | -3.107 | -129.237 |
| 2-aminophenol degradation | -488.655 | -130.701 |

| | Control surface I (Rubber elastomer) | Artificial biofilm |
|---|---|---|
| 1.2-dichloroethane degradation | -201.191 | -165.107 |
| L-isoleucine biosynthesis IV | -148.034 | -168.044 |
| vanillin and vanillate degradation II | -2.725 | -215.080 |
| biphenyl degradation | -3.587 | -217.544 |
| diphenyl ethers degradation | -3.627 | -219.984 |
| Rubisco shunt | -239.832 | -233.873 |
| fatty acid biosynthesis initiation II | -3.286 | -257.094 |
| nitrate reduction I (denitrification) | -3.427 | -288.755 |
| heptadecane biosynthesis | -390.095 | -304.322 |
| adenosylcobalamin salvage from cobinamide II | -14.646 | -304.830 |
| putrescine degradation I | -22.569 | -308.553 |
| sulfide oxidation II (sulfide dehydrogenase) | -1.700 | -323.906 |
| melibiose degradation | -3.135 | -715.451 |
